# Effects of vitro sucrose on quality components of tea plants (*Camellia sinensis*) based on transcriptomic and metabolic analysis

**DOI:** 10.1186/s12870-018-1335-0

**Published:** 2018-06-18

**Authors:** Yumei Qian, Shuxiang Zhang, Shengbo Yao, Jinxin Xia, Yanzhi Li, Xinlong Dai, Wenzhao Wang, Xiaolan Jiang, Yajun Liu, Mingzhuo Li, Liping Gao, Tao Xia

**Affiliations:** 1 State Key Laboratory of Tea Plant Biology and Utilization, Anhui Agricultural University, 130 West Changjiang Rd, Hefei, 230036 Anhui China; 2 School of Biological and Food Engineering, Suzhou University, 49 Middle Bianhe Rd, Suzhou, 234000 Anhui China; 3 School of Life Science, Anhui Agricultural University, 130 West Changjiang Rd, Hefei, 230036 Anhui China

**Keywords:** *Camellia sinensis*, Polyphenol biosynthesis, Volatile, Sucrose induction, Transcriptomic and metabolic analysis

## Abstract

**Background:**

Tea plants [*Camellia sinensis* (L.) O. Kuntze] can produce one of the three most widely popular non-alcoholic beverages throughout the world. Polyphenols and volatiles are the main functional ingredients determining tea’s quality and flavor; however, the biotic or abiotic factors affecting tea polyphenol biosynthesis are unclear. This paper focuses on the molecular mechanisms of sucrose on polyphenol biosynthesis and volatile composition variation in tea plants.

**Results:**

Metabolic analysis showed that the total content of anthocyanins, catechins, and proanthocyanidins(PAs) increased with sucrose, and they accumulated most significantly after 14 days of treatment. Transcriptomic analysis revealed 8384 and 5571 differentially expressed genes in 2-day and 14-day sucrose-treated tea plants compared with control-treated plants. Most of the structural genes and transcription factors (TFs) involved in polyphenol biosynthesis were significantly up-regulated after 2d. Among these transcripts, the predicted genes encoding glutathione S-transferase (GST), ATP-binding cassette transporters (ABC transporters), and multidrug and toxic compound extrusion transporters (MATE transporters) appeared up regulated. Correspondingly, ultra-performance liquid chromatography-triple quadrupole mass spectrometry (UPLC-QQQ-MS/MS) analysis revealed that the content of non-galloylated catechins and oligomeric PAs decreased in the upper-stem and increased in the lower-stem significantly, especially catechin (C), epicatechin (EC), and their oligomeric PAs. This result suggests that the related flavonoids were transported downward in the stem by transporters. GC/MS data implied that four types of volatile compounds, namely terpene derivatives, aromatic derivatives, lipid derivatives, and others, were accumulated differently after in vitro sucrose treatment.

**Conclusions:**

Our data demonstrated that sucrose regulates polyphenol biosynthesis in *Camellia sinensis* by altering the expression of transcription factor genes and pathway genes. Additionally, sucrose promotes the transport of polyphenols and changes the aroma composition in tea plant.

**Electronic supplementary material:**

The online version of this article (10.1186/s12870-018-1335-0) contains supplementary material, which is available to authorized users.

## Background

The tea plant [*Camellia sinensis* (L.) O. Kuntze] is one of the most important economic crops cultivated in China, Japan, India, and other countries. Its leaves are used for making the tea beverage, one of three most widely consumed non-alcoholic beverages around the world because it contains abundant polyphenols, theanine, caffeine, and other secondary metabolites [1]. Among them, the polyphenol, also called tea polyphenol, is a collective term for phenolic acids and flavonoids including flavanols (catechins), anthocyanins, PAs (also named condensed tannins), and other special derivatives. Polyphenols account for 18–36% of the dry weight of tender leaves and are responsible for tea’s flavor [2–4]. Some studies have suggested that polyphenols play crucial roles in plant stress resistance. For example, they are crucial for protecting the tea plant against pathogens and insects [5, 6]. Additionally, polyphenols are the main functional ingredient in tea for preventing cancer, cardiovascular diseases, and obesity [7].

Studies have indicated that polyphenol biosynthesis in plants is influenced by chemical and physical factors, such as nutrients, hormones, and environmental conditions [8–13]. Among them, sucrose acts not only as carbon source for energy storage and sugar transportation, but also as a signal involved in metabolic processes such as anthocyanin synthesis in plants [14, 15]. Since the late twentieth century, the effects of sucrose on flavonoid and anthocyanin biosynthesis in grapes and radishes have been studied [16–18]. In *Arabidopsis thaliana*, sucrose induces anthocyanin biosynthesis through the upregulation of structural genes and positive transcription factors involved in the flavonoid biosynthesis pathway and potentially also through the concurrent down-regulation of the negative transcription factor, MYB-LIKE 2 (MYBL2) [19–21]. Previous studies also reported that sucrose could act as a signaling molecule, by first activating PRODUCTION OF ANTHOCYANIN PIGMENT 1 (PAP1) expression by a sucrose-specific signaling pathway and then triggering the expression of structural genes involved in anthocyanin and flavonoid biosynthesis [14, 19, 22, 23]. The sucrose-specific signaling pathway may be activated by different disaccharides, such as sucrose, maltose, and their breakdown products (glucose and fructose); however, sucrose is the most effective inducer of anthocyanin biosynthesis in Arabidopsis [23]. Liu et al. reported sucrose induction increases the content of non-galloylated catechins and up-regulates the expression of putative genes involved in their biosynthetic pathway in both tea callus and seedling [24]. Additionally, Wang et al. also reported sucrose up-regulates the expression of *Camellia SINENSIS* FLAVONOID 3′5′-HYDROXYLASE (CsF3′5′H), an important branch point gene involved in catechins biosynthesis [25]. In this study, test-tube tea plantlets were used to test for testing the effects of sucrose on polyphenol biosynthesis after 2, 7, 14, and 28d treatments. The results indicated that sucrose can increase the expression of structural genes involved in the biosynthesis of anthocyanins, catechins, and procyanidins. The sucrose specific induction machenism in tea plant is still unclear, one important reason is that we lack the information supported by accurate genome annotations.

Next-Generation Sequencing (NGS) based on the Illumina Hiseq 2000 platform provides a fast, cost-effective, and reliable approach to acquire abundant transcripts, especially for non-model organisms without reference genomic sequences [26]. In tea plants, the NGS technology has been used for analysis of putative genes associated with tea quality and stress response [27–29]. Here, it was performed to investigate the molecular mechanism of sucrose on polyphenol biosynthesis in tea plants and to provide a comprehensive analysis of the network of biochemical and cellular processes responding to sucrose.

In addition, we determined whether in vitro sucrose treatment affects the production of volatiles—the second group of compounds that affect tea taste and flavor in addition to polyphenols.

## Results

### Effects of sucrose on polyphenol accumulation

**Fig. 1:**
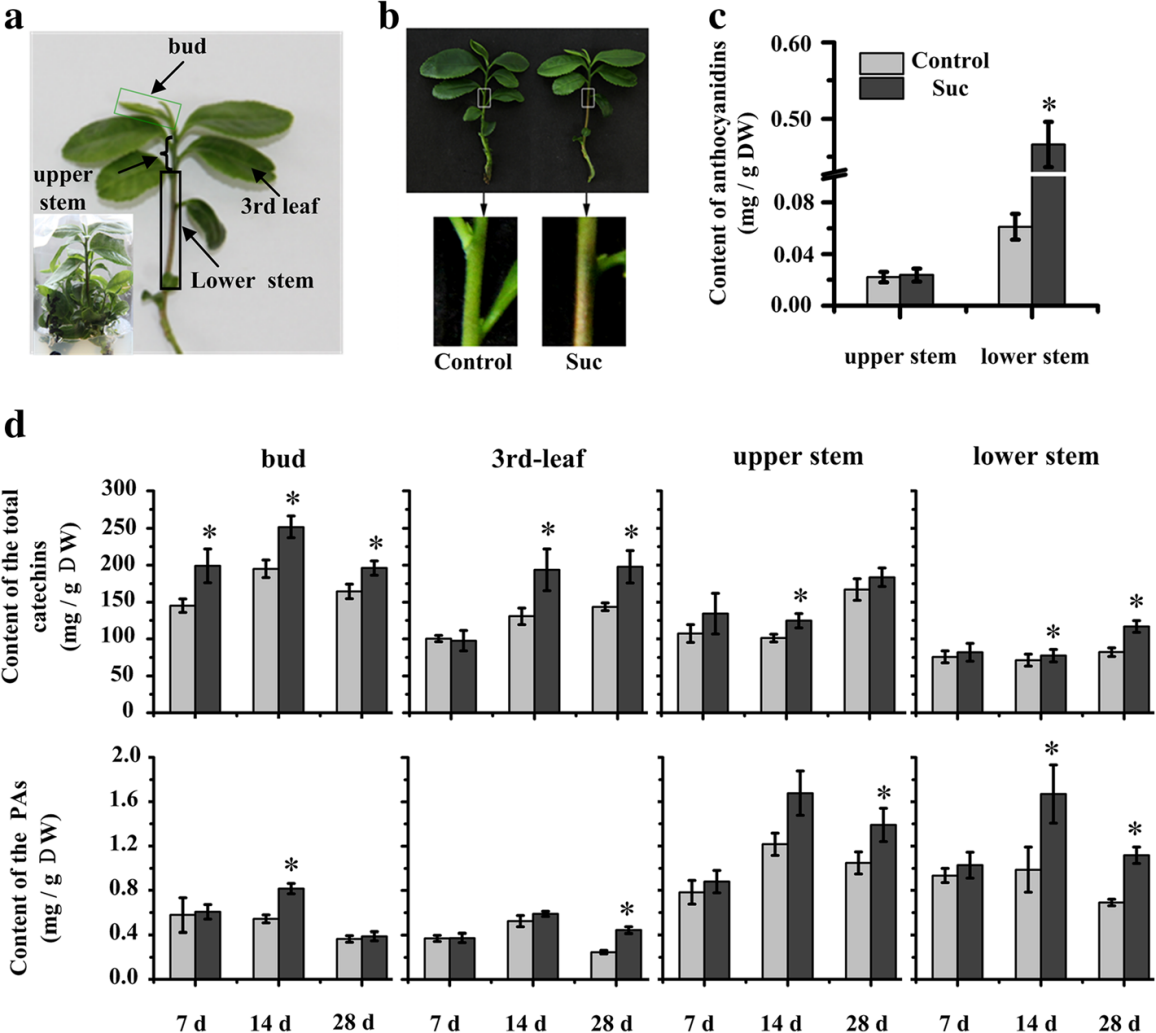
Effects of sucrose on polyphenol accumulation in test-tube tea plantlets. **a**. Test-tube tea plantlets; **b**. Red pigments accumulated in stems of plantlet after feeding sucrose; **c**. Anthocyanin levels are significantly different in the lower part of the stem; **d**. Accumulation of total catechins and PAs in various organs after 7, 14 and 28 d sucrose treatment. Note: * indicates significance at *P* < 0.05. The data represents the mean value of three biological replicates

Similar sized test-tube tea plantlets were cultured on Murashige and Skoog standard medium (MS, Control) and MS supplemented with 90 mM sucrose (MS + 90 mM sucrose, Suc) for 28d (Fig. 1a). The stem of the plantlets grown on Suc for 9-14d began to turn red (Fig. 1b), while no red pigmentation was observed in the stem of the plantlets grown on MS or MS supplemented with 90 mM mannitol (data not shown). The anthocyanin levels were significantly different only in the lower part of the stem and were 7-fold higher than that in the control (Fig. 1c). Furthermore, the accumulation of total catechins and PAs in various organs of tea plants is affected by sucrose (Fig. 1d). The effects of sucrose treatment on polyphenol accumulation were observed after 7 and 14 days of treatment (Fig. 1d). However, the effects of sucrose on total catechins and PAs accumulation were not observed at 2d treatment (data not shown).

Polyphenol, including phenolic acids, catechin monomers, oligomeric PAs, and flavonols, in different tissues of tea plantlets after 14d treatment was quantitatively measured using UPLC-QQQ-MS/MS (Table 1). Three types of phenolic acids were measured, including quinic acid, gallic acid derivatives (β-glucogallin, galloyl acid and galloylquinic acid), and hydroxycinnamic acid derivatives (caffeoylquinic acid and p-coumaroylquinic acid). The effect of sucrose on compound accumulation was different. For example, sucrose increased the content of galloylquinic acid, a special phenolic acid in the tea plant, increased in most parts of the plants, except for in the bud. However, the content of β-glucogallin, the precursor of galloylated catechins, significantly decreased by 84% in buds and by 71% in upper stems [30]. Monomers of flavanols (catechins) can be classified into non-galloylated and galloylated catechins and mainly exist in buds and upper stems. More non-galloylated catechins accumulated in buds and lower stems after sucrose treatment; however, their content in upper stems decreased significantly. Catechin (C) and epicatechin (EC) decreased by 69% in upper stems. The galloylated catechin content in buds and lower stems was not affected by sucrose, and its content in the 3rd leaf and upper stem decreased by 19%. Seven types of oligomeric PAs accumulated in the bud and 3rd leaf. Their content in lower stems increased 3-fold. However, their content in upper stems significantly decreased after sucrose treatment. For example, B2 (an oligomeric C or EC), decreased by 81%. The content of flavonols in the tea plant was also affected by sucrose. Among them, the flavonol with di-hydroxyl groups on the B-ring was significantly affected by sucrose, and its amount decreased by almost 40% in the third leaf and upper stems and by 14% in buds. However, its content increased by 1-fold in the lower stem.

**Table 1:** Effects of sucrose on polyphenol accumulation in different tissues of tea plantlets after 14d treatment using UPLC-QQQ-MS/MS

Compound	Control	Suc	ratio	Control	Suc	ratio	Control	Suc	ratio	Control	Suc	ratio
	bud	bud		3rd leaf	3rd leaf		up-stem	up-stem		down-stem	down-stem	
Phenolic acid (mg/g)												
Quinic acid	44.21 ± 2.01	86.06 ± 4.05	1.95	6.55 ± 0.23	7.45 ± 0.35	1.14	39.43 ± 1.89	40.19 ± 1.70	1.02	3.72 ± 0.15	6.19 ± 0.29	1.67
Gallic acid derivatives												
β-glucogallin	9.42 ± 0.41	1.47 ± 0.11	0.16	0.90 ± 0.05	0.97 ± 0.05	1.08	2.83 ± 0.12	0.81 ± 0.03	0.29	0.02 ± 0.00	0.01 ± 0.00	0.73
galloyl acid	0.38 ± 0.01	0.36 ± 0.02	0.95	0.08 ± 0.00	0.10 ± 0.01	1.19	0.25 ± 0.01	0.16 ± 0.01	0.65	0.20 ± 0.01	0.03 ± 0.00	0.13
galloylquinic acid	14.09 ± 0.9	13.29 ± 0.7	0.94	0.13 ± 0.01	0.47 ± 0.05	3.76	3.55 ± 0.16	6.71 ± 0. 32	1.89	0.09 ± 0.00	0.11 ± 0.01	1.15
Summation	23.88 ± 1.32	15.12 ± 0.83	0.63	1.11 ± 0.06	1.54 ± 0.11	1.40	6.64 ± 0.29	7.68 ± 0.36	1.16	0.31 ± 0.01	0.14 ± 0.01	0.47
Hydroxycinnamic acids derivatives												
caffeoylquinic acid	0.16 ± 0.01	0.14 ± 0.01	0.90	0.14 ± 0.01	0.02 ± 0.00	0.17	0.12 ± 0.01	0.06 ± 0.00	0.52	ND	ND	
p-coumaroylquinic acid	2.29 ± 0.12	3.44 ± 0.15	1.51	ND	0.51 ± 0.02		0.45 ± 0.04	2.11 ± 0.13	4.65	ND	ND	
Summation	2.45 ± 0.13	3.59 ± 0.16	1.47	0.14 ± 0.01	0.53 ± 0.02	3.93	0.57 ± 0.05	2.17 ± 0.13	3.78	ND	ND	
Flavanols (mg/g)												
NongalloylatedCatechins												
catechin	2.79 ± 0.12	3.74 ± 0.16	1.34	0.86 ± 0.04	2.59 ± 0.13	3.02	5.51 ± 0.26	1.71 ± 0.08	0.31	0.99 ± 0.04	3.03 ± 0.13	3.06
epicatechin	3.64 ± 0.21	6.26 ± 0.29	1.72	3.37 ± 0.15	3.81 ± 0.19	1.13	8.73 ± 0.31	2.75 ± 0.11	0.31	3.02 ± 0.13	4.47 ± 0.15	1.48
gallocatechin	1.00 ± 0.06	2.66 ± 0.12	2.66	1.54 ± 0.08	2.30 ± 0.11	1.49	1.43 ± 0.06	1.34 ± 0.07	0.93	0.24 ± 0.01	1.05 ± 0.06	4.36
epigallocatechin	13.91 ± 0.8	26.89 ± 1.20	1.93	10.47 ± 0.62	7.93 ± 0.38	0.76	13.99 ± 0.80	12.11 ± 0.71	0.87	4.24 ± 0.15	3.19 ± 0.14	0.75
Summation	21.34 ± 1.19	39.55 ± 1.77	1.85	16.23 ± 0.89	16.63 ± 0.71	1.02	29.67 ± 1.36	17.90 ± 0.97	0.60	8.49 ± 0.33	11.74 ± 0.48	1.38
Galloylatedcatechins												
epicatechingallate	22.38 ± 1.09	20.75 ± 1.01	0.93	3.82 ± 0.15	3.58 ± 0.15	0.94	11.08 ± 0.84	9.29 ± 0.83	0.84	2.18 ± 0.13	2.05 ± 0.98	0.94
epigallocatechingallate	89.03 ± 4.21	95.88 ± 4.67	1.08	18.29 ± 0.95	14.26 ± 0.68	0.78	52.19 ± 2.65	42.19 ± 2.05	0.81	6.30 ± 0.31	6.46 ± 0.31	1.03
Summation	111.40 ± 5.30	116.63 ± 5.68	1.05	22.11 ± 1.10	17.84 ± 0.83	0.81	63.27 ± 3.49	51.48 ± 2.88	0.81	8.47 ± 0.44	8.51 ± 1.29	1.00
total Catechins	132.74 ± 6.49	156.18 ± 7.45	1.18	38.34 ± 1.99	34.47 ± 1.85	0.90	92.94 ± 4.85	69.38 ± 3.85	0.75	16.97 ± 0.77	20.25 ± 1.77	1.19
Proanthocyanidins (area)												
m/z 865	ND	ND		ND	ND		ND	ND		490 ± 36	6057 ± 312	12.36
m/z 577 PAs B2	33,626 ± 1670	52,158 ± 2600	1.55	17,040 ± 850	36,819 ± 1830	2.16	122,564 ± 6115	23,153 ± 1160	0.19	37,345 ± 1876	155,893 ± 7805	4.17
m/z 729EC-ECG	17,582 ± 880	18,560 ± 930	1.06	2125 ± 105	3947 ± 185	1.86	15,214 ± 755	7597 ± 380	0.50	2089 ± 117	10,067 ± 515	4.82
m/z 593EC-EGC or ECDG	2300 ± 110	6507 ± 320	2.83	5361 ± 260	17,280 ± 855	3.22	16,556 ± 815	5454 ± 267	0.33	3475 ± 184	20,418 ± 1015	5.88
m/z 761EGC-EGCG	11,308 ± 565	21,097 ± 1050	1.87	3841 ± 180	4698 ± 225	1.22	6627 ± 325	9111 ± 438	1.37	909 ± 55	4932 ± 264	5.43
m/z 745	3570 ± 178	5468 ± 270	1.53	2062 ± 105	3916 ± 185	1.90	3806 ± 185	3133 ± 148	0.82	ND	3992 ± 196	
m/z 609(EGC-EGC)	3809 ± 190	11,528 ± 570	3.03	11,219 ± 550	32,040 ± 1505	2.86	11,924 ± 585	5195 ± 246	0.44	2501 ± 129	17,566 ± 868	7.02
flavonols derivatives (area)												
tri-hydroxyl in B-ring												
myricetin 3-O-galactoside	3929 ± 203	5100 ± 268	1.30	705 ± 42	ND		ND	2367 ± 123		ND	269 ± 12	
myricetin 3-O- glucoside	6797 ± 346	6940 ± 359	1.02	1220 ± 58	1301 ± 72	1.07	3577 ± 185	3404 ± 164	0.95	ND	260 ± 10	
Summation	10,726 ± 549	12,040 ± 627	1.12	1925 ± 100	1301 ± 72	0.68	3577 ± 185	5771 ± 287	1.61	ND	529 ± 30	
di-hydroxyl in B-ring												
quercetin 3-O-galactosylrutinoside	2539 ± 136	2235 ± 126	0.88	780 ± 48	489 ± 34	0.63	2465 ± 131	1025 ± 55	0.42	806 ± 45	684 ± 28	0.85
quercetin3-O-glucosylrutinoside	9680 ± 496	8675 ± 456	0.90	3933 ± 208	2379 ± 126	0.60	5641 ± 291	3847 ± 184	0.68	793 ± 45	2704 ± 136	3.41
quercetin 3-galactoside	1404 ± 87	1367 ± 78	0.97	428 ± 30	ND		1376 ± 62	674 ± 31	0.49	290 ± 18	208 ± 12	0.72
quercetin 3-O-glucoside	2465 ± 138	1630 ± 89	0.66	911 ± 42	850 ± 45	0.93	1284 ± 58	783 ± 45	0.61	168 ± 7	526 ± 32	3.14
Summation	16,088 ± 857	13,907 ± 749	0.86	6052 ± 328	3717 ± 201	0.61	10,766 ± 542	6330 ± 315	0.59	2056 ± 115	4122 ± 208	2.00
mono -hydroxyl in B-ring												
kaempferol-3-O-galactosylrutinoside	338,752 ± 16,950	290,468 ± 14,530	0.86	61,932 ± 3085	39,007 ± 1968	0.63	137,928 ± 6870	130,099 ± 6485	0.94	23,498 ± 1164	18,979 ± 1001	0.81
kaempferol3-O-glucosylrutinoside	853,325 ± 42,664	753,945 ± 37,665	0.88	206,694 ± 10,345	120,862 ± 6055	0.58	316,408 ± 15,808	334,177 ± 16,675	1.06	23,691 ± 1135	37,778 ± 1982	1.59
kaempferol-3-O-galactoside	ND	933 ± 59		154 ± 10	287 ± 28	1.86	447 ± 31	484 ± 30	1.08	ND	255 ± 10	
kaempferol-3-O-glucoside	ND	20,072 ± 1008		1491 ± 85	ND		6994 ± 350	9054 ± 446	1.29	496 ± 71	199 ± 9	0.40
Kaempferol-3-O-rhamnosylgalactoside	25,559 ± 1289	26,315 ± 1315	1.03	11,173 ± 560	5296 ± 276	0.47	9333 ± 456	11,450 ± 564	1.23	1567 ± 76	2223 ± 124	1.42
Summation	1,217,636 ± 60,903	1,091,733 ± 54,577	0.90	281,445 ± 14,085	165,452 ± 8327	0.59	471,109 ± 23,515	485,263 ± 24,200	1.03	49,252 ± 2396	59,434 ± 3126	1.21
total flavonols	1,244,449 ± 62,309	1,117,680 ± 55,953	0.90	289,422 ± 14,513	170,470 ± 8600	0.59	485,453 ± 24,242	497,364 ± 24,802	1.02	51,309 ± 2511	63,556 ± 3334	1.24

Note: ND indicates that the polyphenol was not detected; the data represents the mean value of three biological replicates

Digit indicates the ratio of Suc / Control

### Effects of sucrose on volatile compounds

Four types of volatile compounds were measured using GC/ MS, including terpene derivatives, aromatic derivatives, lipid derivative and other compounds, the effect of sucrose on their accumulation was different (see Additional file 1: Table S1). For example, the content of α-farnesene belonging to sesquiterpenoid diterpenoid increased 5.77-fold; the expression of one transcript (Unigene 46,443), which was predicted as the key biosynthetic gene encoding farnesene synthase, was significantly upregulated 3-fold after 2 and 14 days of sucrose treatment (see Additional file 2: Table S2). Here, 33 terpene derivatives were detected and classified into monoterpenoid sesquiterpenoid diterpenoid; these compounds were biosynthesized via methylerythritol phosphate (MEP) and mevalonate (MVA) pathways (see Additional file 3: Figure S1). The expression of HMGR (CL12062.Contig1) and DXS (Unigene57617) and DXR (Unigene46601) as the key genes involving in terpenoid backbone pathway were up-regulated by sucrose. The expression of one transcript (CL1850.Contig3 encoding linalool synthase) was not significantly affected by sucrose; and the content of linalool and geraniol in tea leaf only decreased by 4%. Additionally, the expression of 1 transcript (Unigene9305 encoding (E)-nerolidol synthase) was up-regulated by sucrose after 2d; however, its expression was down- regulated by sucrose after 14d; and the content of the (E)-nerolidol only decreased by 5%.

### Effects of sucrose on the expression of key structural genes related to polyphenol biosynthesis using qRT-PCR

**Fig. 2:**
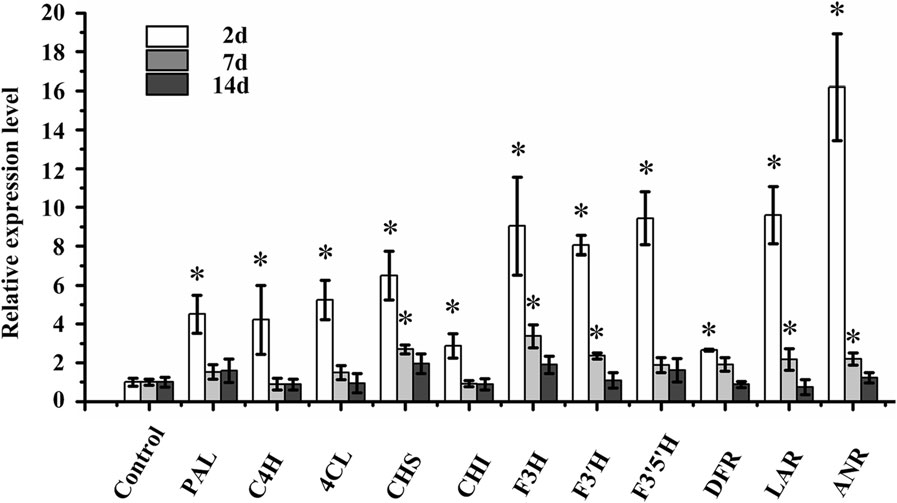
Effects of sucrose on expression of key structural genes involved in polyphenol biosynthesis using qRT-PCR. Note: * indicates significance with |log2 Ratio| ≥ 1. The data represents the mean value of three biological and three technical replicates

For further analysis of the effects of sucrose on polyphenol biosynthesis at the transcriptional level, Quantitative real-time-PCR (qRT-PCR) was used to test the expression of 11 key structural genes involved in the polyphenol biosynthetic pathway (Fig. 2). Their expression significantly increased 3-fold after 2d treatment. After 7d, the expression of Chalcone synthase (CHS), Flavanone 3-hydroxylase (F3H), Flavonoid 3′-hydroxylase (F3′H), Leucoanthocyanidin reductase (LAR), and Anthocyanidin reductase (ANR) increased 1-fold. After 14d, the effect of sucrose on the above genes was less noticeable.

### Sequencing, de novo assembly, and functional annotation

To obtain the overall transcriptional levels of genes in the tea plant treated by sucrose after 2 and 14d, four normalized cDNA libraries (2d: 2nd D Control and Suc; 14d: 14th D Control and Suc) were constructed for transcriptome sequencing. Based on the Illumina Hiseq 2000 platform, 21,381,193,620 nucleotide (nt) bases were generated from all libraries in total and about 237.6 million clean reads (94.94% of the raw reads) were achieved for de novo assembly (see Additional file 4: Table S3). Finally, a total of 118,843 transcripts were obtained with an average length of 1212 nt and a N50 of 1999 nt (see Additional file 5: Table S4).

To predict the functions of the assembly transcripts, a total of 82,459 transcripts (69.38% of all assembled Unigenes) were annotated using the NR (Non-redundant protein database), NT (Non-redundant nucleotide database), Swiss-Prot (Annotated protein sequence database), KEGG (Kyoto encyclopedia of genes and genomes), COG (Clusters of orthologous groups of protein), and GO (Gene ontology) databases based on two levels of sequence similarity, sequence-based and domain-based alignments, with an e-value<1e-5 (see Additional file 6: Table S5).

### Analysis of DEGs responding to sucrose

**Fig. 3:**
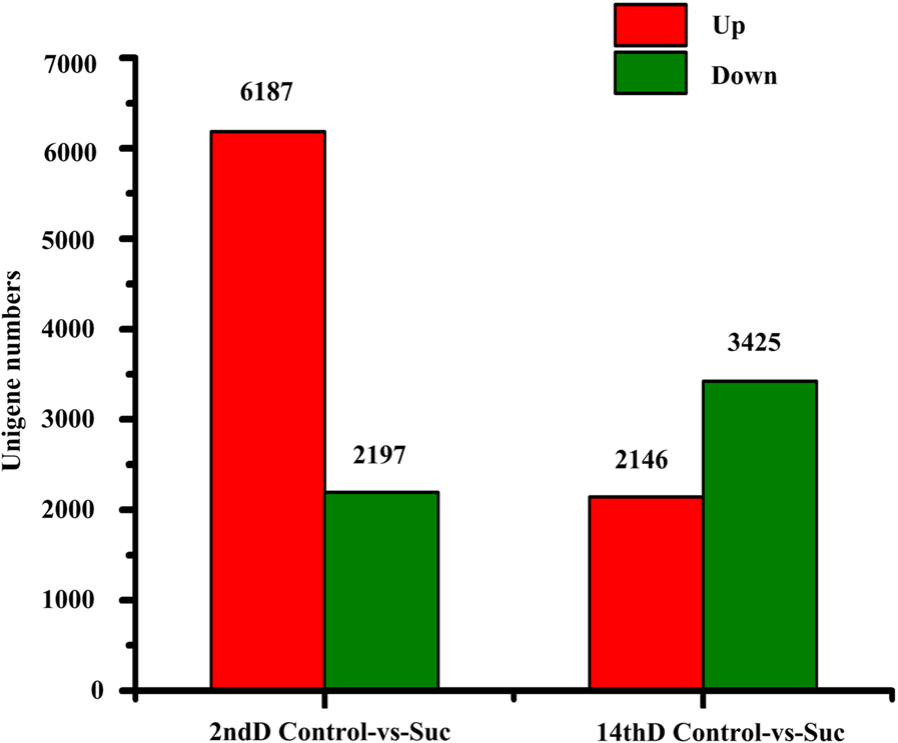
Statistics of DEGs from tea plants responding to sucrose. Note: DEGs were classified into two classes; the red bar indicates up-regulated and the green bar indicates down-regulated, the digit indicates the number of DEGs

Using the fragments per kb per million reads (FPKM) method, the DEGs between two samples were identified with a significant threshold of |log2 Ratio (FPKM Control-vs-Suc) | ≥ 1 and the false discovery rate (FDR) of ≤0.001 based on the *P*-value threshold set as ≤1e-5. A total of 8384 DEGs were detected in 2nd D Control-vs-Suc. Among them, 6187 DEGs (73.80% of the total DEGs) were up-regulated. A total of 5571 DEGs were detected in 14th D Control-vs-Suc, and only 2146 DEGs (38.52% of the total DEGs) were up-regulated (see Fig. 3).

### GO function and KEGG pathways analysis of DEGs responding to sucrose

To better understand the biological functions of DEGs responding to sucrose, GO and KEGG analyses were performed for comparisons of 2nd D Control-vs-Suc and 14th D Control-vs-Suc. GO functional enrichment analysis indicated that 49 and 48 GO terms were classified into three ontologies which changed significantly between 2nd D and 14th D Control-vs-Suc (see Additional file 7: Figure S2).

**Fig. 4:**
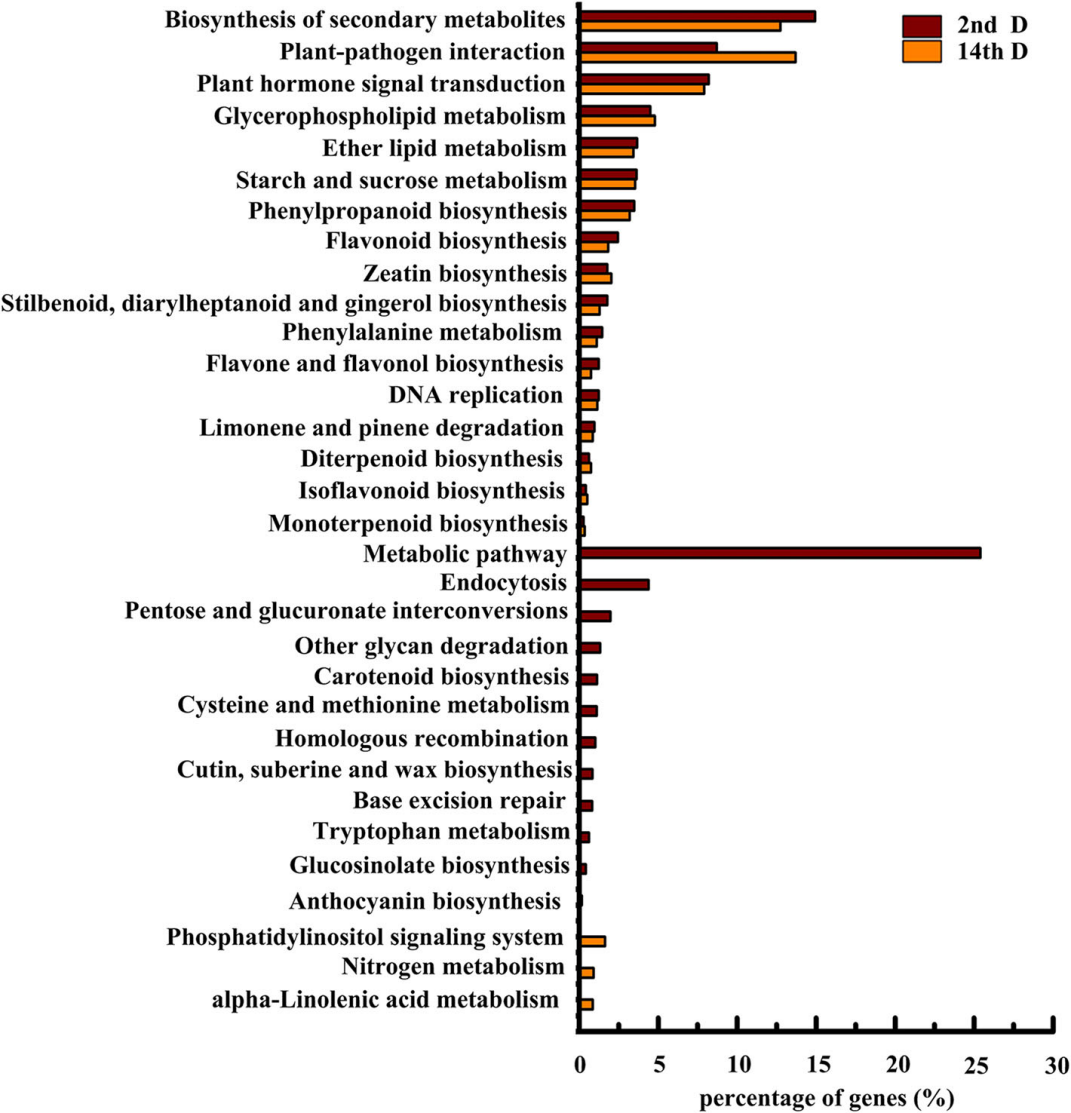
The pathways significantly enriched by DEGs after 2d and 14d sucrose treatment. Note: the horizontal coordinates indicate percent of DEGs, the vertical coordinates indicate significantly enriched pathways of differentially expressed genes

A total of 3553 DEGs (7.46% of all the transcripts aligned to the KEGG database) were annotated and 29 KEGG pathways were enriched significantly in the 2nd D Control-vs-Suc comparison based on a Q-value of ≤0.05. Among them, the most enriched pathway was “flavonoid biosynthesis” (Table 2). In 14th D Control-vs-Suc comparison, 2009 DEGs (4.22% of all the transcripts aligned to KEGG databases) were annotated and 20 KEGG pathways were significantly enriched with the same threshold. The most enriched pathway was that for “plant-pathogen interaction” (Table 3). A total of 17 KEGG-enriched pathways were common between second and fourteenth D Control-vs-Suc. Of the 12 KEGG pathways specific to the second D Control-vs-Suc comparison, one was the KEGG-enriched pathway for anthocyanin biosynthesis (Fig. 4).

**Table 2:** Gene ontology analysis of DEGs obtained from tea plants treated by sucrose after 2d

	Pathway	DEGs genes	All genes	Q-value
		(3553)	(47655)	
1	Flavonoid biosynthesis	87 (2.45%)	314 (0.66%)	2.35E-25
2	Biosynthesis of secondary metabolites	530 (14.92%)	4746 (9.96%)	1.33E-20
3	Phenylpropanoid biosynthesis	124 (3.49%)	653 (1.37%)	1.76E-20
4	Stilbenoid, diarylheptanoid and gingerol biosynthesis	63 (1.77%)	233 (0.49%)	3.38E-18
5	Flavone and flavonol biosynthesis	44 (1.24%)	165 (0.35%)	1.41E-12
6	Phenylalanine metabolism	52 (1.46%)	234 (0.49%)	1.76E-11
7	Plant hormone signal transduction	291 (8.19%)	2615 (5.49%)	4.76E-11
8	Zeatin biosynthesis	63 (1.77%)	365 (0.77%)	5.88E-09
9	Cutin, suberine and wax biosynthesis	30 (0.84%)	116 (0.24%)	1.65E-08
10	Pentose and glucuronateinterconversions	70 (1.97%)	452 (0.95%)	6.26E-08
11	DNA replication	44 (1.24%)	244 (0.51%)	4.79E-07
12	Carotenoid biosynthesis	40 (1.13%)	212 (0.44%)	4.95E-07
13	Limonene and pinene degradation	34 (0.96%)	170 (0.36%)	1.05E-06
14	Metabolic pathways	902 (25.39%)	10,454 (21.94%)	1.79E-06
15	Ether lipid metabolism	130 (3.66%)	1142 (2.4%)	8.47E-06
16	Starch and sucrose metabolism	129 (3.63%)	1141 (2.39%)	1.24E-05
17	Diterpenoid biosynthesis	22 (0.62%)	105 (0.22%)	6.04E-05
18	Tryptophan metabolism	22 (0.62%)	107 (0.22%)	7.84E-05
19	Other glycan degradation	47 (1.32%)	328 (0.69%)	8.46E-05
20	Endocytosis	156 (4.39%)	1526 (3.2%)	2.40E-04
21	Glycerophospholipid metabolism	160 (4.5%)	1577 (3.31%)	2.69E-04
22	Glucosinolate biosynthesis	15 (0.42%)	64 (0.13%)	3.18E-04
23	Isoflavonoid biosynthesis	15 (0.42%)	72 (0.15%)	1.25E-03
24	Plant-pathogen interaction	309 (8.7%)	3440 (7.22%)	1.60E-03
25	Monoterpenoid biosynthesis	10 (0.28%)	41 (0.09%)	3.38E-03
26	Anthocyanin biosynthesis	6 (0.17%)	20 (0.04%)	1.26E-02
27	Cysteine and methionine metabolism	40 (1.13%)	339 (0.71%)	1.27E-02
28	Base excision repair	29 (0.82%)	228 (0.48%)	1.51E-02
29	Homologous recombination	36 (1.01%)	323 (0.68%)	4.46E-02

**Table 3:** Gene Ontology analysis of DEGs obtained from tea plants treated by sucrose after 14d

	Pathway	DEGs genes	All genes	Q-value
		(2009)	(47655)	
1	Plant-pathogen interaction	275 (13.69%)	3440 (7.22%)	3.78E-23
2	Phenylpropanoid biosynthesis	64 (3.19%)	653 (1.37%)	3.04E-08
3	Zeatin biosynthesis	41 (2.04%)	365 (0.77%)	6.03E-07
4	Flavonoid biosynthesis	37 (1.84%)	314 (0.66%)	6.41E-07
5	Plant hormone signal transduction	159 (7.91%)	2615 (5.49%)	5.74E-05
6	Stilbenoid, diarylheptanoid and gingerol biosynthesis	26 (1.29%)	233 (0.49%)	1.37E-04
7	Biosynthesis of secondary metabolites	256 (12.74%)	4746 (9.96%)	3.87E-04
8	Diterpenoid biosynthesis	15 (0.75%)	105 (0.22%)	5.25E-04
9	Glycerophospholipid metabolism	96 (4.78%)	1577 (3.31%)	3.06E-03
10	DNA replication	23 (1.14%)	244 (0.51%)	3.55E-03
11	Phenylalanine metabolism	22 (1.1%)	234 (0.49%)	4.47E-03
12	alpha-Linolenic acid metabolism	17 (0.85%)	164 (0.34%)	5.98E-03
13	Starch and sucrose metabolism	71 (3.53%)	1141 (2.39%)	7.18E-03
14	Isoflavonoid biosynthesis	10 (0.5%)	72 (0.15%)	7.18E-03
15	Limonene and pinene degradation	17 (0.85%)	170 (0.36%)	7.18E-03
16	Monoterpenoid biosynthesis	7 (0.35%)	41 (0.09%)	1.12E-02
17	Ether lipid metabolism	69 (3.43%)	1142 (2.4%)	1.39E-02
18	Nitrogen metabolism	18 (0.9%)	203 (0.43%)	1.68E-02
19	Phosphatidylinositol signaling system	33 (1.64%)	465 (0.98%)	1.74E-02
20	Flavone and flavonol biosynthesis	15 (0.75%)	165 (0.35%)	2.62E-02

### Effects of sucrose on polyphenol biosynthesis based on transcriptome sequencing

**Fig. 5:**
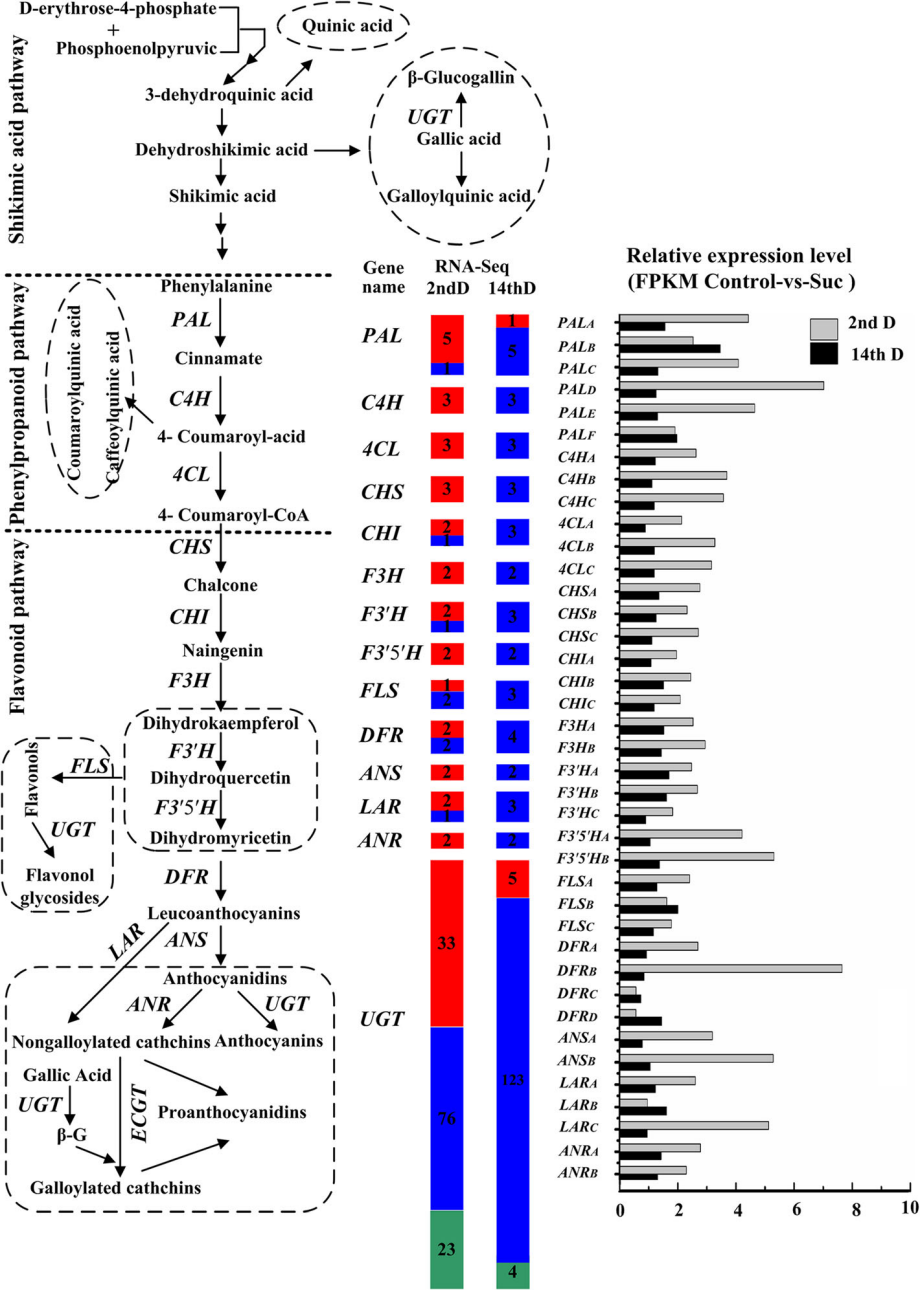
Effects of sucrose on the expression of structural genes related to polyphenol biosynthesis in tea plants after 2d and 14d. Note: Red indicates significant up-regulation, blue indicates no difference, green indicates significant down-regulation. Digit indicates the number of Unigenes

Based on the ratio of FPKM Control-vs-Suc, most of the transcripts involved in the phenylpropanoid and flavonoid pathways were up-regulated 2-fold or more after 2d of treatment. Additionally, the expression of transcripts annotated as Phenylalanine ammonialyase (PAL), Dihydroflavonol 4-reductase (DFR), LAR, and Anthocyanidin synthase (ANS) was notably up-regulated. After 14 days of treatment, the expression of only PALB increased 1-fold, whereas others were not affected by sucrose (Fig. 5). These results indicate that tea polyphenol biosynthesis is comprehensively affected by sucrose.

### Effects of sucrose on the expression of transcription factors involved in polyphenol biosynthesis based on transcriptome sequencing

Polyphenol biosynthesis in plants is regulated by transcription factors (TFs) including R2R3-MYB, bHLH, and WD40 [31, 32]. In this study, 37 DEGs were predicted to be MYB members and were classified into three types: R1 (4 DEGs), R2R3 (29 DEGs), and R1R2R3 (4 DEGs). Most DEGs (23/37) were up-regulated after sucrose treatment for 2 days, and only five DEGs were up-regulated after sucrose treatment for 14 days (Table 4). Additionally, the phylogenetic tree, including 29 R2R3-MYBs and 126 Arabidopsis R2R3-MYBs, were classified into 13 subgroups (see Additional file 8: Figure S3). Phylogenetic analysis indicated that 33 bHLHs were dispersed into 15 subfamilies (see Additional file 9: Figure S4), and 21 of them were up-regulated after sucrose treatment for 2d (Table 5).

**Table 4:** Analysis of DEGS-predicted as R2R3-MYB obtained from tea plants treated by sucrose

Gene ID	Gene	2ndD	14thD	Type	Subgroups	Putative function clade and gene function
	length	fold	fold		No.	
CL5525.Contig4	955	476.9^a^	–	R2R3	other	Trichome development-regulated: AtMYB82 [69]
Unigene18972	1084	17.02^a^	0.41^b^	R1R2R3	Unknown	
Unigene35962	3506	13.97^a^	0.49^b^	R1R2R3	Unknown	
Unigene12085	975	13.54^a^	0.32^b^	R2R3	6	Anthocyanin biosynthes-related: AtMYB75and AtMYB90 [54, 70, 71]
Unigene41846	938	4.98^a^	–	R2R3	6	Secondary cell wall formation-related: AtMYB75 [72]
Unigene35958	3304	6.28^a^	–	R1R2R3	Unknown	
CL8695.Contig1	1179	5.47^a^	–	R2R3	5	Seed pigmentation biosynthesis -controlled: AtMYB123 [48, 73]
Unigene11002	1229	2.93^a^	–	R2R3	5	
Unigene7972	1143	5.41^a^	–	R2R3	9	Seed germination and reproductive development-related AtMYB17 [74, 75]
CL1441.Contig4	2364	2.85^a^	–	R2R3	9	Petal development: AtMYB16 [76]
						Repressor of cell outgrowth: AtMYB106 [77]
Unigene24177	714	4.91^a^	–	R2R3	other	
Unigene20350	1829	2.20^a^	–	R2R3	other	
CL12359.Contig1	3219	2.56^a^	–	R2R3	other	
CL5017.Contig2	1322	4.04^a^	0.34^b^	R2R3	1	Hypersensitive response: AtMYB30Cooperates with BES1 to regulate
CL8708.Contig1	1933	2.91^a^	–	R2R3	1	brassinosteroid-induced gene Expression; abiotic stress response, SA–mediated pathway AtMYB30 [77]
Unigene13855	767	3.84^a^	–	R2R3	15	Epidermal cell fate specification: AtMYB23 [78] Trichome development: AtMYB0 and AtMYB23,
CL7877.Contig1	887	3.25^a^	–	R2R3	15	Root hair patterning-controlled AtMYB66 [79]
Unigene1868	527	2.68^a^	–	R1	Unknown	
Unigene16731	1118	2.41^a^	–	R2R3	14	Axillary meristem initiation in roots-related: AtMYB36 [80]
CL3134.Contig13	4926	2.40^a^	–	R1R2R3	Unknown	
CL13057.Contig1	995	2.31^a^	–	R2R3	4	The battle against UV by repressing C4H: AtMYB4 [81]
CL13057.Contig2	827	–	2.64^a^	R2R3	4	
CL2339.Contig1	1129	2.24^a^	–	R2R3	21	Lignin, xylan and cellulose biosynthesis-regulated: AtMYB52, AtMYB54 and AtMYB69 [82]
						Ovule and fruit development: AtMYB117 [83]
						ABA hypersensitivity and drought tolerance: AtMYB52 [84]
CL8255.Contig3	1314	–	2.02^a^	R2R3	7	Flavonol glycosides-related: AtMYB11, AtMYB12 and AtMYB111 [34]
CL6408.Contig3	1494	2.01^a^	–	R2R3	2	Shoot apex morphogenesis: AtMYB13 [85]
CL9344.Contig1	1068	–	0.25^b^	R2R3	2	Cold stress tolerance: AtMYB14 and AtMYB15 [86, 87]
CL6408.Contig1	1557	–	0.45^b^	R2R3	2	
CL5350.Contig2	1322	–	0.16^b^	R2R3	2	
Unigene48919	574	0.41^b^	–	R2R3	2	
CL1581.Contig2	1552	–	0.18^b^	R1	Unknown	
CL7764.Contig2	980	–	0.15^b^	R1	Unknown	
Unigene6794	537	–	2.47^a^	R2R3	other	
Unigene36358	1700	–	2.01^a^	R2R3	other	AS1 leaf morphogenesis (polarity specificity) and plant immune response: AtMYB91 [88];
						Rough-sheath development: AtMYB91 [89]
Unigene11308	1618	–	2.10^a^	R2R3	13	Stomatal closure: AtMYB61 [90];
						Multiple aspects of plant resource allocation-controled: AtMYB61 [91]
Unigene38120	1427	–	0.47^b^	R2R3	22	Stomatal closure-regulated: AtMYB44,AtMYB70, AtMYB73 and AtMYB77 [92, 93]
						Auxin signaling pathway- modulated: AtMYB77 [94];
Unigene39226	735	0.49^b^	–	R2R3	20	GA metabolism and signaling involved in regulation starvation responses:AtMYB62 [95];
						Cell separation processes-related: AtMYB116 [96]
Unigene2945	935	0.44^b^	–	R1	Unknown	

Note: “^a^”indicates significant up-regulation; “–”indicates no difference; “^b^”indicates significant down-regulation. Unknown and other indicate Unigene is not grouped

**Table 5:** Analysis of DEGS-predicted as bHLH obtained from tea plants treated by sucrose

GeneID	Gene	2ndD	14thD	Subfamily	Gene name	Putative function clade and gene function
	length	fold	fold	No.	in *Arabidopsis*	
Unigene60798	496	1967.8^a^	–	3	AtbHLH18	
Unigene26720	1512	15.20^a^	–		AtbHLH25	
CL2783.Contig8	2320	280.50^a^	–	25	AtbHLH74	Regulation root growth: AtbHLH74 [97]
CL4342.Contig3	2304	2.02^a^	–			
CL9935.Contig2	1894	7.50^a^	0.42^b^	25	AtbHLH137	
Unigene21382	845	4.85^a^	–	25	AtbHLH63	
Unigene29122	545	8.35^a^	2.14^a^	1	AtbHLH33	Cold tolerance: AtbHLH33,AtbHLH116(ICE1),AtbHLH61and AtbHLH93 [98]
					AtbHLH116	Stomatal differentiation: AtbHLH33(ICE2)and AtbHLH116 [99];
					AtbHLH61	Drought stress:AtbHLH116(ICE1) [100]
					,AtbHLH93	
CL1034.Contig1	3358	–	0.30^b^	1	AtbHLH35	
CL1034.Contig2	889	–	0.27^b^		AtbHLH27	Drought stress:bHLH27 [100]
CL1034.Contig5	942	–	0.27^b^		AtbHLH29	Iron Uptake-regulated:AtBHLH29 [101]
CL1768.Contig1	648	4.33^a^	–	10	AtbHLH57,	
					AtbHLH67,	
					AtbHLH70	
CL12543.Contig1	1074	3.58^a^	–	10	AtbHLH71	
CL9545.Contig2	1190	2.38^a^	–	10	AtbHLH94	
CL9545.Contig1	813	2.31^a^	–		AtbHLH96	
Unigene17438	326	2.29^a^	–			
CL13089.Contig1	2067	0.37^b^	–	10	AtbHLH57	
Unigene32633	1085	3.54^b^	–	9	AtbHLH91	
					AtbHLH10	
					AtbHLH89	
Unigene10835	1585	0.34^b^	–	26	AtbHLH69	Female gametophyte development;
					AtbHLH66	Response to phosphate deficiency stress:AbHLH69, AbHLH66 [53]
Unigene2520	732	2.89^a^	–	16	AtbHLH135	
Unigene5385	844	2.74^a^	–	5	AtbHLH42	Anthocyanin biosynthesis (GL3, EGL3, TT8) [53]
Unigene21617	2490	2.35^a^	–			Regulate proanthocyanidin biosynthesis [49, 51]
Unigene23312	1076	2.49^a^	–	13	AtbHLH106	Abiotic stress-involved in cold, salt, ABA and drought stress:
					AtbHLH107	AtbHLH106 [102]
Unigene47124	874	2.47↑	0.43^b^	27	AtbHLH128,	
Unigene39259	789	–	0.00^b^		AtbHLH129	Regulation root elongation and ABA response:AtbHLH129 [103]
					AtbHLH80	
					AtbHLH81	
					AtbHLH122	Drought and osmotic stress tolerance, ABA catabolism repression: AtbHLH122 [100]
					AtbHLH130	
Unigene28617	886	2.23^a^	–	15	AtbHLH133	
					AtbHLH68	
CL8951.Contig3	2042	–	0.30^b^	15	AtbHLH123	
Unigene38437	809	2.20^a^	–	19	AtbHLH149	
CL496.Contig1	889	2.19^a^	–	31	AtbHLH140	
Unigene20853	1750	–	2.87^a^	31	AtbHLH87	Flower and fruit development, initiation/maintenanceofaxillary meristems [53]
CL2917.Contig5	3168	–	0.28^b^	2	AtbHLH3	Male fertility-affected:AtbHLH3(JAM3) [104]
Unigene63328	1505	–	4.65^a^	2	AtbHLH14	
CL10048.Contig2	1395	–	0.05^b^	7	AtbHLH92	Tolerance to NaCl and osmotic stresses: bHLH92 [105]
CL1061.Contig1	2440	–	0.10^b^	7	AtbHLH41	

Note:“^a^”indicates significant up-regulation; “–”no difference; “^b^” indicates significant down-regulation

**Fig. 6:**
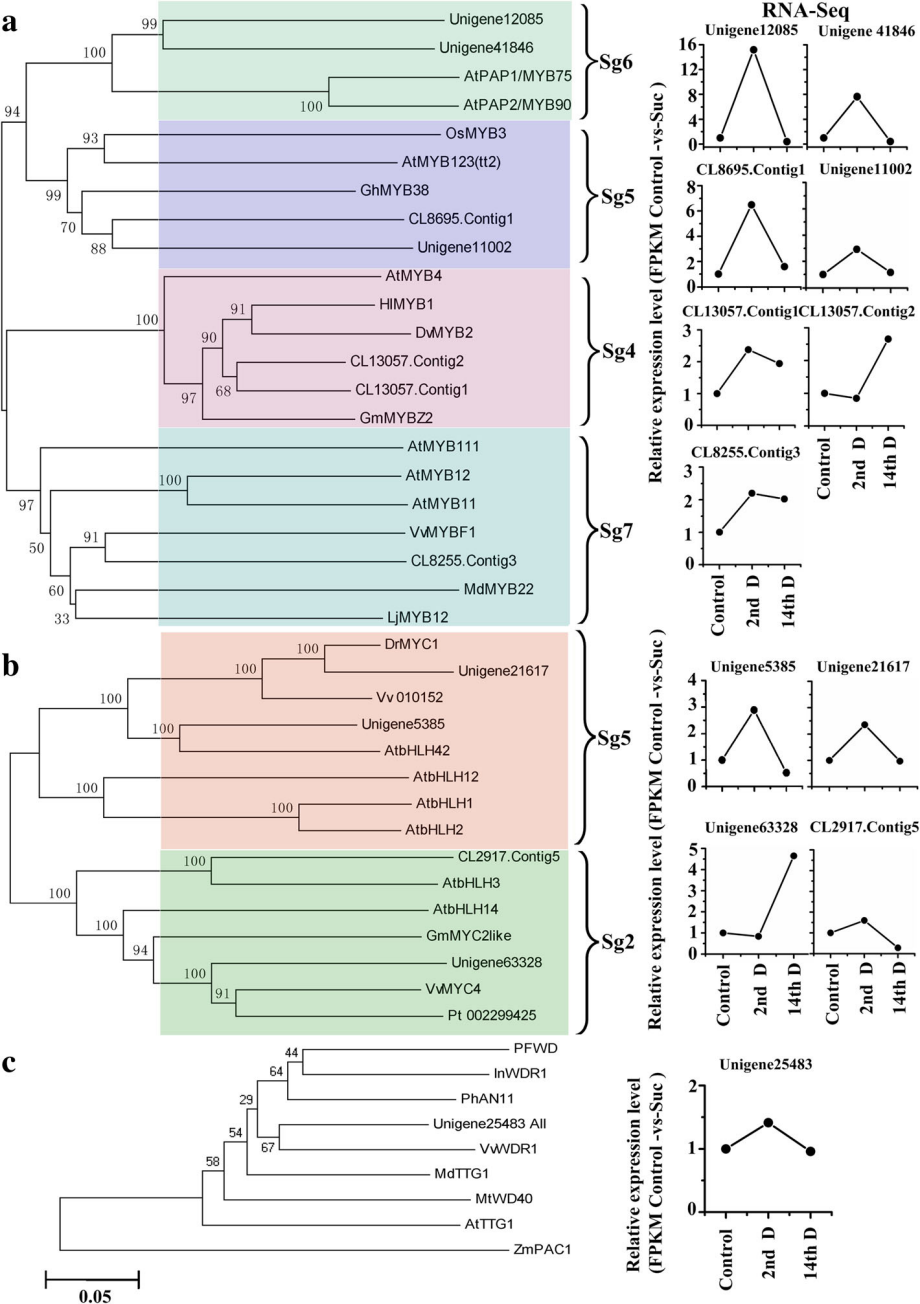
Effects of sucrose on the expression of R2R3-MYB (**a**), bHLH (**b**) and WD40 (**c**) involved in polyphenol biosynthesis. Note: The phylogenetic tree was constructed based on amino acid sequences using MEGA5 according to the neighbor-joining method. GenBank accession numbers: MYB-Sg4: AtMYB4 (AEE86955), HlMYB1 (CAI46244), DvMYB2 (BAJ33514), GmMYBZ2 (ABI73970); MYB-Sg5: OsMYB3 (BAA23339), AtMYB123 (Q9FJA2), GhMYB38 (AAK19618); MYB-Sg6: AtMYB75 (AEE33419), AtMYB90 (AEE34503); MYB-Sg7: AtMYB11 (XP_002876680), AtMYB12 (O22264), AtMYB111 (XP_002865729), VvMYBF1 (ACV81697), MdMYB22 (AAZ20438), LjMYB12 (BAF74782). bHLH-Sg5: AtbHLH12 (Q8W2F1), AtbHLH42 (Q9FT81), AtbHLH1 (Q9FN69), AtbHLH2 (Q9CAD0), DrMYC1 (AEC03343), Vv_010152 (CAN62848.1); bHLH-Sg2: AtbHLH3 (O23487), AtbHLH14 (O23090), GmMYC2like (XP_003528771), VvMYC4 (XP_002279973), Pt_002299425 (XP_002299425). WD40: PFWD (BAB58883), InWDR (BAE94407), PhAN11 (AAC18914), VvWDR1 (NP_001268101), MdTTG1 (ADI58760), AtTTG1 (CAB45372), ZmPAC1 (AAM76742)

The R2R3-MYBs, bHLH, and WD40 TFs, could act as regulators of polyphenol biosynthesis individually or jointly. The R2R3-MYBs in Subgroup (Sg) 4 and Sg7 were predicted to be negative and positive regulators, respectively, for controlling the production of flavonols via regulating the up-stream genes involved in polyphenol biosynthetic pathway [33, 34]. However, the R2R3-MYBs in Sg5 and Sg6 require both bHLH (subfamily 2, 5, and 24) and WD40 for construction into a ternary complex MYB-bHLH-WD40 (MBW) for positively regulating down-stream genes involved in polyphenol biosynthetic pathway [31, 35, 36]. Here, 7 DEGs were classified into the above mentioned 4 subgroups of R2R3-MYBs. After 2d sucrose treatment, the expression of 3 DEGs (Unigene12085, Unigene 41,846 and CL8695 Contig1) in Sg6 and Sg5 were significantly up-regulated 6-fold; and the expression of CL13057.Contig2 in Sg4 was down-regulated significantly (Fig. 6a). Additionally, 2 DEGs (Unigene 21,617, Unigene 5385) in Subfamily 5 of bHLHs were up-regulated by sucrose (Fig. 6b). Based on the same method, only one transcript (Unigene25483) was predicted to be involved in the MBW complex, and its expression was not affected by sucrose (Fig. 6c).

### Effects of sucrose on the expression of genes involved in polyphenol transport

**Fig. 7:**
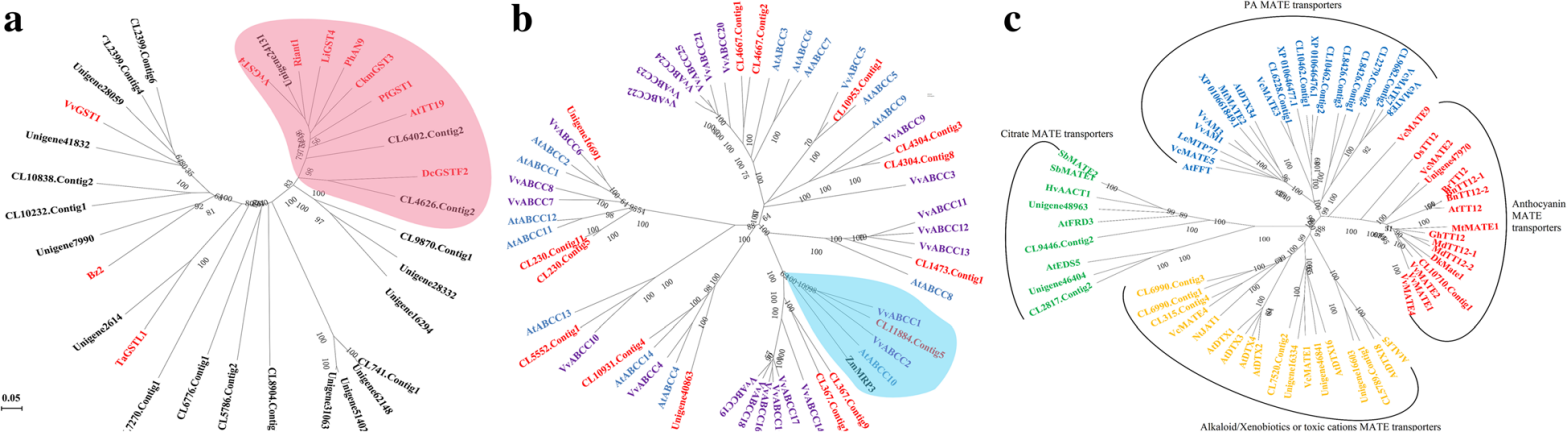
Effects of sucrose on the expression of Unigenes encoding transporters related to flavonoid. **a**. Glutathione S-transferase;**b**. ABC transporters;**c***. mate* transporters. Note: The phylogenetic tree was constructed based on amino acid sequences using MEGA5 according to the neighbor-joining method. All protein sequences used in this figure were provided in Additional file 13: Txt S1

In plants, transporters (ABCs and MATEs), and GSTs are involved in polyphenol transporting. These transporters are found in many species including Arabidopsis TT19 and TT12 genes (AtTT19; AtTT12), the grape GST and ABCC1 genes (VvGST19; VvABCC1), the maize MRP3 gene (ZmMRP3), and the *Medicago truncatula* MATE (MtMATE) [37–42]. In the present study, 22, 15, and 21 DEGs were predicted to encode GST, ABC, and MATE-transporters, respectively. Phylogenetic analysis showed three transcripts closely corresponding to the above 3 transporters (Fig. 7). Among them, the expression of the ABC (CL11884.Contig7) and MATE (Unigene47970) decreases significantly by sucrose after 2d, and their expression increases after 14d (Additional file 10: Table S6). However, the expression of the GST (Unigene24131) responds to sucrose opposite of the above mentioned two transcripts (Additional file 10: Table S6). The above results indicate there could be different transporters and GSTS for transporting the polyphenol in tea plants.

### Using qRT-PCR for transcriptome sequencing validation

To validate the results of transcriptome sequencing, 30 DEGS were randomly selected to be analyzed by qRT-PCR. We found that 83.33% of the total transcripts expression was consistent with the results from transcriptome sequencing, including 11 genes involved in polyphenol biosynthesis. Detailed information regarding the selected DEGs and 11 genes is presented in Additional file 11: Figure S5.

## Discussion

### The mechanisms of sucrose effects on tea polyphenol biosynthesis

In the past decades, exploration of tea polyphenol biosynthesis and their influencing factors have become a hotspot for research in plant secondary metabolism [30, 43]. Due to self-incompatibility, rich genetic diversity, and the large genome in tea plants, little genomic information is available and the molecular mechanisms of tea polyphenol biosynthesis are still unclear [44, 45]. Our previous research demonstrated tea polyphenol shared a similar biosynthetic pathway to other plants, such as shikimic acid, phenylpropanoid, and flavonoids synthetic pathways [2]. Its biosynthesis is also affected by sucrose, light, and other factors [24, 46].

Studies have demonstrated sucrose-specific transcriptional regulation of polyphenol biosynthesis in plants. For example, Boss et al. reported that the expression of DFR involved in anthocyanin and PAs biosynthesis in grape was induced by sucrose treatment, and they speculated that the accumulation of the two metabolites in grape berry skin could be attributed to sugar accumulation during grape berry development [47]. According to microarray data, it was revealed that anthocyanin biosynthesis in Arabidopsisis is stimulated by sucrose which acts as a signal to activate PAP1, a TF for activating the expression of structural genes involved in anthocyanin biosynthetic pathway, such as PAL, Cinnamate 4-hydroxylase (C4H), 4-coumaroyl-CoA ligase (4CL), and others [19, 23]. However, the structural gene F3′5′H and transcriptional factor PAP2 are not affected by sucrose [19]. In tea plants, Wang et al. found the expression of Cs F3′5′H increased 15-fold by feeding sucrose [25]. Liu et al. reported that sucrose induced the accumulation of catechins and upregulated the expression of putative genes involved in their biosynthetic pathway [24]. In this study, the total content of catechins and PAs significantly increases by sucrose induction for 7d and the accumulation of anthocyanin increases 7-fold in the stems of tea plantlets after 14d sucrose treatment. Only after 2d treatment, the expression of structural genes involved in their biosynthesis is up-regulated based on qRT-PCR and transcriptome sequencing. After 14d, the effects of sucrose were not detected.

In Arabidopsis, the correct expression of BANYULS (BAN) as a key gene of PAs biosynthesis is necessary for activation of TT2 (AtMYB123, an R2R3-MYB TF encoded by the TRANSPARENT TESTA2 gene) and TT8 (AtbHLH42, a bHLH TF encoded by the TRANSPARENT TESTA8 gene) together with TTG1 (AtTTG1, a WD-repeat protein encoded by the TRANSPARENTTESTA GLABRA1gene) [48–50]. TT2 cannot be replaced by any other AtMYB [51]. Additionally, the genes of Sg4, 5, 6, and 7 R2R3-MYB and the Subfamily2, 5, and 24 bHLH are all involved in flavonoid biosynthesis [35, 52]. Based on their amino acid sequence alignment, it was found that 7 R2R3-MYB and 4 bHLH are predicted to participate in flavonoid biosynthesis in tea plants [53]. In the present study, seven DEGs were classified into the aforementioned four subgroups of the R2R3-MYBs and four DEGs into bHLH subfamilies 5 and 2. Among them, the expression of 3 transcripts (Unigene12085, Unigene41846, and CL8695.Contig1) in R2R3-MYB Sg6 and Sg5 were up-regulated 6-fold; this finding is consistent with those of studies indicating that sucrose can induce the expression of PAP1/MYB75, which is essential for sucrose-induced anthocyanin biosynthesis [19, 23, 48, 54]. In addition, Unigene5385 corresponded to TT8 and its expression was significantly increased by sucrose treatment for 2d, indicating that it might be involved with others in regulating the accumulation of anthocyanins and PAs [55, 56]. Notably, only one transcript (Unigene25483) corresponds closely to AtTTG1, consistent with the results reported in *C. sinensis* [53]. However, it was not affected by sucrose, possibly because WD40 proteins have no catalytic activity and act as docking platforms for MYB and bHLH proteins in regulating flavonoid biosynthesis [48, 51, 53, 57].

As described above, it is inferred the accumulation of tea polyphenol might be directly due to high expression of their structural genes which could be synergistically regulated by TFs.

### The mechanisms of sucrose effects on tea polyphenol transport

Based on analysis of UPLC-QQQ-MS/MS, the non-galloylated catechins and oligomeric PAs were significantly induced by sucrose in bud, 3rd leaf, and lower stems after 14d treatment; however, their content in upper stems decreased significantly, especially C, EC, and their oligomeric PAs. This suggests there was flavonoid transport in tea plants. Extensive research shows GST, ABC, and MATE transporters could be involved in flavonoid transport and there are at least three mechanisms, GST-linked, Vesicle trafficking (VT), and MATE transporters [38, 39, 42, 58–61]. In the present study, only three transcripts annotated as GST, ABC, and MATE were involved in flavonoid transport, and their expression was differently affected by sucrose. As described above, it is inferred that there are varieties of proteins for synergistically transporting tea polyphenol in tea plants. However, the molecular mechanisms remain unclear.

### Impact of sucrose on the volatile

It is known that the flavor of tea is basically determined by taste (non-volatile compounds) and aroma (volatile compounds) [62]. The tea polyphenol is crucial for tea taste, and the terpene derivatives including monoterpenoid and sesquiterpenoid are important aroma ingredient due to their delectable fruit fragrance and low detection threshold [63]; for example, linalool and geraniol have fruity and sweet floral scents [62]. Previous research indicated that linalool, geraniol, nerolidol, ionone, and jasmone were identified as odour-active in many types of green teas [64, 65]. In the present study, (Z)-jasmone and β-ionone content increased by 2.63 and 0.57-fold, respectively; however, linalool, geraniol and nerolidol were not significantly affected by sucrose. As the biosynthetic pathway volatile compounds is complicated, and the molecular mechanisms involving in volatile compounds affected by sucrose need to be further studied.

## Conclusions

In this paper, the test-tube tea plantlets were used for investigating the effects of sucrose on polyphenol biosynthesis. Metabolomics and transcriptomics analyses indicated that sucrose up-regulation of anthocyanins, catechins, and PAs biosynthesis. Sucrose controls the expression of structural and regulating genes. Additionally, sucrose promotes the transport of polyphenol in *Camellia sinensis* by the predicted transporters GST, ABC, and MATE involved in polyphenol transport. In summary, these results and analyses present valuable resources for better understanding the biosynthesis molecular mechanisms underlying the main characteristics of secondary metabolites in the tea plant and help improve the nutritional quality of tea.

## Methods

### Plant materials and cultivation conditions

The test-tube tea plantlets [*Camellia sinensis* (L.) O. Kuntzevar. cultivar Nongkangzao] were initially grown in vitro on classical solid MS medium and then transferred to solid MS supplemented with 90 mM sucrose for sucrose feeding studies with 10 h of light (42 μmol/m^2^ s) at 24 ± 1 °C. Correspondingly, similar sized test-tube tea plantlets were transferred to classical solid MS medium for the control under the same conditions. In the above experiments, the tea plantlets were incubated on MS supplemented with 90 mM mannitol for the osmotic control.

For metabolic analysis of polyphenol, the samples of different organs (the buds, third leaves, and the upper and lower stems) were collected from the tea plantlets cultivated after 2, 7, 14, and 28d. Meanwhile, samples of leaves were also collected from the tea plantlets cultivated after 2, 7, 14 and 28d for analysis of polyphenol biosynthesis at the transcriptional level. All the collected samples were immediately frozen in liquid nitrogen and stored at − 80°Cuntil use. In this study, approximately 10 independent tea plants were collected for one biological replicate; and three biological replicates were used for analysis.

### Chemicals and reagents

The compounds viz., quinic acid, β-glucogallin, galloyl acid, galloylquinic acid, caffeoylquinic acid, p-coumaroylquinic acid, catechin, epicatechin, gallocatechin, epigallocatechin, epicatechingallate, epigallocatechingallate, procyanidin B_2_, myricetrin, quercitrin, and kaempferitrinwere obtained from Sigma (St Louis, MO, USA) and Axxora Co. and Ltd. (Lausanne,Switzerland). Cyanidin chloride was procured from Axxora Co. and Ltd. (Lausanne, Switzerland). HPLC grade acetic acid, methanol, and acetonitrile were bought from Tedia Co., Ltd. (Fairfield, OH, USA). Concentrated hydrochloric acid, vanillin, and other solvents used for extraction were acquired from Sinopharm Chemical Reagent Co., Ltd. (Shang-hai, China).

### Extraction and quantitative analysis of the polyphenol

Extraction and quantitative analysis of the polyphenol was performed with UPLC-QQQ-MS/MS as suggested by Jiang et al. [2]. The total catechins were extracted and quantitatively analyzed using 1% vanillin–HCl (*w*/*v*) according to the methods described by Wang et al. [66].

Spectrophotometry analysis of anthocyanins was carried out as described by Pang et al. and the molar absorbance of cyanidin-3-*O*-glucoside was used for calculating the total anthocyanin concentration [67].

The total PAs were extracted and quantitatively analyzed using spectrophotometry by the methods reported by Jang et al. and their concentration was converted by using a standard curve of procyanidin B_2_ [2].

### Extraction and analysis of the volatile compounds

Extraction and analysis of the volatile compounds collected from the samples of the leaves of tea plantlets cultivated after14 d were performed with a headspace-solid phase microextraction (HS-SPME) fiber, coupled with gas chromatography (Agilent 7697A) and mass spectrometry (Agilent 7890A) (GC/MS). In brief, 0.3 g of leaves samples were cut up and put in the 20 ml headspace bottle 4 mL by adding boiling double distilled water dissolved 0.8 g KCl. After incubation for 1.5 min, the volatile compounds were collected using a 50/30 μm DVB/CAR/PDMS SPME fiber (Supelco, PA, USA) for 50 min at 70 °C and then desorbed into the GC injection port at 250 °C for 5 min. Subsequently, the volatile compounds were resolved by BD-5 capillary column (30 m × 0.25 mm × 0.25 μm, Agilent) for GC/MS analysis according to Han et al. [64].

### RNA extraction and qRT-PCR analysis

Total RNA was extracted as described by Zhao et al. [53]. The RNA concentration, quality, and integrity were measured by using spectrophotometry (Agilent2100) and gel electrophoresis. The single-stranded complementary deoxyribonucleic acid (cDNA) was synthesized using Prime-Script™ (Takara, Dalian, Code: DRR037A) for qRT-PCR analysis. All the primer sequences were designed using Primer Premier 6.0 and the selected Unigene IDs are detailed in the additional file (see Additional file 12: Table S7). The qRT-PCR assays were performed by using a CFX96™ optical reaction module (Bio-RAD, USA) and the detailed detection system was the same as previously described by Zhao et al. [53]. The resultant relative expression values were normalized against the housekeeping gene glyceraldehyde-3-phosphate dehydrogenase (*GAPDH*) and evaluated from the mean value of three biological and three technical replicates by the 2^-ΔΔCT^ method [68].

### Library construction, RNA-seq and de novo assembly

Library Construction and de novo assembly were performed by Beijing Genome Institute (BGI; Shenzhen, China). Briefly, the specific operations are summarized as follows: the mRNA isolated from the total RNA was fragmented into smaller pieces to create templates for synthesizing the first-strand cDNA. Using the first-strand cDNA as templates, the double-stranded cDNA was produced with random primers (Japan, Takara). Subsequently, these cDNA fragments were processed by end repair using DNA polymerase and polynucleotide kinase and ligation of adapters to produce approximately 200 bp fragments. Finally, these fragments were purified by using Qiaquick Gel Extraction Kit (Qiagen) and enriched with PCR to construct cDNA libraries.

In this study, four cDNA libraries (2d: 2nd D Control and Suc; 14d: 14th D Control and Suc) were examined by using the Agilent 2100 Bioanalyzer and were sequenced using Illumina HiSeq™ 2000. The clean reads were obtained from the raw reads by removing the low-quality reads and the reads with adaptors or unknown nucleotides larger than 5%. Based on assembly of clean reads separately, Unigenes were the resulting sequences after removing redundancy and short contigs separately using the short reads assembling program–Trinity.

### Bioinformatics analysis of the assembled Unigenes

By using BLASTx (E-value 10^− 5^) against the database of NR, NT, GO, Swiss-Prot, COG, and KEGG, the assembled Unigenes were annotated for functional analysis and their expression levels were calculated by the fragments per kb per million reads (FPKM). Differentially expressed genes (DEGs) were identified with a significant threshold of|log2 Ratio of FPKM (Control-vs-Suc)| ≥ 1 and FDR ≤ 0.001 based on the *P*-value threshold set as ≤1e^− 5^. Based on FDR ≤ 0.05, KEGG Pathway analysis was performed to ascertain the main biochemical and signal transduction pathways of DEGs.

### Phylogenetic analysis of transcription factors and transport proteins involved in polyphenols

The phylogenetic trees for transcription factors and transport proteins were constructed according to the method as described by Zhao et al. [53]. Briefly, the MEGA 5.0 software was used for the phylogenetic analysis and the neighbor-joining statistical method was carried out based on amino acid sequences. The Bootstrap method with 1000 replicates was performed for evaluating the tree nodes. By using the p-distance method, evolutionary distances were computed. All the sequences used for the alignment were retrieved from The Arabidopsis Information Resource (TAIR, Carnegie Institution for Science Department of Plant Biology, USA), the UniProt Database (UniProt, Switzerland), and the National Center for Biotechnology Information (NCBI, USA).

## Availability of supporting data

The transcriptome sequencing data based on the Illumina Hiseq 2000 platform obtained from leaves of *Camellia sinensis*are available in NCBI SRA (https://www.ncbi.nlm.nih.gov/sra/ with accessions SRR5427581,SRR5427580,SRR5427578 and SRR5427577.

## Additional files

Additional file 1: **Table S1. **Effects of sucrose on volatile compounds in leaves of tea plants using GC/ MS. Note: The data represents the mean value of three biological replications. The red indicates significant up-regulation; green indicates significant down-regulation; blue indicates no difference;. Digit indicates the ratio of Suc / Control. (DOCX 40 kb)

Additional file 2: **Table S2. **Effects of sucrose on the expression of genes related to aroma. (DOCX 26 kb)

Additional file 3: **Figure S1.** The pathway of terpenoids biosynthesis. (TIF 412 kb)

Additional file 4: **Table S3. **Statistics of sequencing output. Note: Q20 percentage is the proportion of nucleotides with quality value larger than 20, N percentage is proportion of unknown nucleotides in clean reads, GC percentage is proportion of guanidine and cytosine nucleotides among total nucleotides. (DOCX 20 kb)

Additional file 5: **Table S4.** Statistics of assembly quality. Note: Total Consensus Sequences represents the all assembled Unigenes, Distinct Clusters represents the cluster Unigenes; the same cluster contains some highly similar (more than 70%) Unigenes and these may come from same gene or homologous gene, Distinct Singletons represents Unigenes from a single gene. (DOCX 21 kb)

Additional file 6: **Table S5.** Summary of Unigenes annotated to six databases. (DOCX 20 kb)

Additional file 7: **Figure S2.** GO functional classification of DEGs obtained from tea plants treated by sucrose after 2d (A) and 14d (B). Note: GO functions are showed on X-axis, the right Y-axis shows the number of DEGs which have the GO function, the left Y-axis shows the percentage of DEGs. (TIF 27083 kb)

Additional file 8: **Figure S3.** Evolutionary relationships of DEGs belong to R2R3-MYB obtained from tea plants treated by sucrose. Note: The phylogenetic tree was constructed based on amino acid sequences using MEGA5 per the neighbor-joining method, digit indicates subgroup, other indicates DEGs are not grouped. (TIF 18963 kb)

Additional file 9: **Figure S4.** Evolutionary relationships of DEGs belong to bHLH obtained from tea plants treated by sucrose. Note: The phylogenetic tree was constructed based on amino acid sequences using MEGA5 according to the neighbor-joining method, digit indicates subfamily. (TIF 16115 kb)

Additional file 10: **Table S6.** All expression data of contigs in Fig. 7. (XLSX 18 kb)

Additional file 11: **Figure S5.** Validation of DEGs obtained from tea plants treated by sucrose using qRT-PCR. A. DEGs obtained from tea plants treated by sucrose after 2d; B. DEGs obtained from tea plants treated by sucrose after 14d. Note: The data of qRT-PCR represents the mean value of three biological and three technical replicates. (TIF 16000 kb)

Additional file 12: **Table S7.** Primers used for qRT-PCR and detailed information regarding the selected DEGs. Note: “↑” indicates significant up-regulation; “–”no difference; “↓”indicates significant down-regulation. (DOCX 39 kb)

Additional file 13: **Txt S1.** Protein sequences used in figure 7. (TXT 117 kb)

## Abbreviations

4CL: 4-coumaroyl-CoA ligase
ABCtransporter: ATP-binding cassette transporter
ANR: Anthocyanidin reductase
ANS: Anthocyanidinsynthase
At: *Arabidopsis thaliana*
C: Catechin
C4H: Cinnamate 4-hydroxylase
cDNA: Single-stranded complementary deoxyribonucleic acid
CHI: Chalconeisomerase
CHS: Chalcone synthase
Cs: *Camellia sinensis*
DEGs: Differentially expressed genes
DFR: Dihydroflavonol 4-reductase
EC: Epicatechin
ECGT: Epicatechin:1-O-galloyl-β-D-glucose-O-galloyltransferase
F3′5′H: Flavonoid3′,5′-hydroxylase
F3′H: Flavonoid 3′-hydroxylase
F3H: Flavanone 3-hydroxylase
FDR: False discovery rate
FLS: Flavonol synthase
FPKM: Fragments per kb per million reads
GAPDH: Glyceraldehyde-3-phosphate dehydrogenase
GST: Glutathione S-transferase
LAR: Leucoanthocyanidinreductase
MATE transporter: Multidrug and toxic compound extrusion transporter
MBW: MYB-bHLH-WD40
MS: Murashige and Skoog standard medium
NGS: The next-generation sequencing
PAL: Phenylalanine ammonialyase
PAP1: Production of anthocyanin pigment 1
PAP2: Production of anthocyanin pigment 2
PAs: Proanthocyanidins
qRT-PCR: Quantitative real-time-PCR
Sg: Subgroup
TF: Transcription factor
TT12: Transparent testa 12
TT19: Transparent testa19
TT2: Transparent testa 2
TTG1: Transparent testa glabra1
UGT: UDPG-glucosyltransferase
UPLC-QQQ-MS/MS: Ultra-performance liquid chromatography-triple quadrupole mass spectrometry
