# Supplementary material for: Effects of vitro sucrose on quality components of tea plants (Camellia sinensis) based on transcriptomic and metabolic analysis
Source: BMC Plant Biol. 2018 Jun 18;18:121. doi: 10.1186/s12870-018-1335-0 (PMC6007066; doi:10.1186/s12870-018-1335-0)
Supplement: Supplementary file 1 — Table S1. Effects of sucrose on volatile compounds in leaves of tea plants using GC/ MS. Note: The data represents the mean value of three biological replications. The red indicates significant up-regulation; green indicates significant down-regulation; blue indicates no difference;. Digit indicates the ratio of Suc / Control. (DOCX 40 kb) [file 12870_2018_1335_MOESM1_ESM.docx]

Supplementary Material：

Table S1 Effects of sucrose on volatile compounds in leaves of tea plants using GC/ MS.

| **Compounds** | **RT (min)** | **CAS** | **Control** | **Suc** | **ratio** |
| --- | --- | --- | --- | --- | --- |
|  |  |  | (area) | (area) |  |
| **Terpene derivatives** |  |  |  |  |  |
| **Monoterpenoid** |  |  |  |  |  |
| 6-methyl-5-Hepten-2-one | 18.6361 | 110-93-0 | 1.08E+05±3.13E+03 | 2.94E+05±1.26E+04 | 2.72 |
| β-Pinene | 18.8559 | 127-91-3 | 4.42E+06±4.18E+04 | 3.51E+06±1.50E+05 | 0.79 |
| β-Myrcene | 18.8560 | 123-35-3 | 2.06E+06±8.64E+04 | 1.70E+06±8.49E+04 | 0.83 |
| 2,6,6-Trimethyl-bicyclo[3.1.1]hept-3-ylamine | 19.2611 | 69460-11-3 | 4.14E+04±2.07E+03 | 7.12E+04±1.78E+03 | 1.72 |
| α-Phellandrene | 19.6418 | 99-83-2 | 1.92E+05±9.42E+03 | 1.31E+05±2.61E+03 | 0.68 |
| (+)-4-Carene | 19.6420 | 29050-33-7 | 2.43E+05±7.06E+03 | 1.64E+05±3.45E+03 | 0.67 |
| 2-Carene | 20.0623 | 554-61-0 | 1.89E+05±8.49E+03 | 1.42E+05±3.40E+03 | 0.75 |
| D-Limonene | 20.5327 | 5989-27-5 | 1.04E+06±6.48E+04 | 8.51E+05±1.79E+04 | 0.82 |
| Limonene | 20.5425 | 138-86-3 | 4.75E+05±1.85E+04 | 3.28E+05±9.85E+03 | 0.69 |
| α-Pinene | 20.6226 | 7785-70-8 | 1.08E+06±4.65E+04 | 8.28E+05±4.14E+04 | 0.77 |
| β-Ocimene | 21.0330 | 13877-91-3 | 3.09E+06±1.30E+05 | 2.22E+06±1.02E+05 | 0.72 |
| (Z)- β-Ocimene | 21.0379 | 3338-55-4 | 1.19E+06±4.87E+04 | 1.05E+06±4.21E+04 | 0.89 |
| Linalool | 23.0649 | 78-70-6 | 6.38E+07±3.60E+06 | 6.15E+07±3.13E+06 | 0.96 |
| Neo-allo-ocimene | 23.9657 | 7216-56-0 | 4.56E+05±1.96E+04 | 3.22E+05±1.10E+04 | 0.71 |
| 4,4-Dimethyl-1,1a,3a,4,5,6- | 24.5411 | 264628-27-5 | 5.69E+04±2.22E+03 | 2.95E+05±8.86E+03 | 5.19 |
| hexahydrocyclopropa[c]pentalene |  |  |  |  |  |
| 7-methyl-3-methylene-6-Octen-1-ol | 26.9332 | 13066-51-8 | 2.98E+05±9.55E+03 | 3.61E+05±5.02E+03 | 1.21 |
| (Z)-Geraniol | 27.1735 | 106-25-2 | 7.00E+05±4.71E+04 | 6.72E+05±3.09E+04 | 0.96 |
| (Z)-iso-Geraniol | 27.2936 | 5944-20-7 | 3.37E+05±1.62E+04 | 3.58E+05±1.75E+04 | 1.06 |
| Myrtenol | 27.7740 | 515-00-4 | 5.48E+04±2.52E+03 | 9.31E+04±3.82E+03 | 1.70 |
| Geraniol | 27.9292 | 106-24-1 | 1.74E+07±1.06E+06 | 1.56E+07±2.00E+05 | 0.90 |
| Citral | 28.4696 | 5392-40-5 | 3.31E+05±2.40E+04 | 3.58E+05±1.45E+04 | 1.08 |
| Dodecanal | 32.4331 | 112-54-9 | 8.13E+04±2.84E+03 | 1.24E+05±3.73E+03 | 1.53 |
| **Sesquiterpenoid** |  |  |  |  |  |
| (E)- Nerolidol | 30.5965 | 40716-66-3 | 2.31E+05±9.01E+03 | 2.20E+05±4.41E+03 | 0.95 |
| Copaene | 31.8422 | 3856-25-5 | 4.30E+04±1.98E+03 | 8.40E+04±3.36E+03 | 1.95 |
| (Z)-Jasmone | 32.1778 | 488-10-8 | 9.96E+04±2.89E+03 | 3.61E+05±1.12E+04 | 3.63 |
| 3-Methyl-2-pent-2-enyl-cyclopent-2-enone | 32.1828 | 1000193-43-6 | 9.50E+04±3.51E+03 | 2.37E+05±8.54E+03 | 2.50 |
| γ-Elemene | 33.1883 | 29873-99-2 | 1.27E+05±3.26E+03 | 1.90E+05±8.77E+03 | 1.50 |
| Geranylacetone | 33.4490 | 689-67-8 | 1.99E+05±8.36E+03 | 3.33E+05±1.63E+04 | 1.67 |
| β-Ionone | 34.4397 | 14901-07-6 | 7.03E+04±2.67E+03 | 1.11E+05±5.53E+03 | 1.57 |
| cis-sesquisabinene hydrate | 34.5247 | 1000374-17-8 | 7.46E+04±2.39E+03 | 7.41E+04±3.34E+03 | 0.99 |
| α-Farnesene | 34.8998 | 502-61-4 | 1.52E+06±5.94E+04 | 1.03E+07±2.06E+05 | 6.77 |
| Cedrol | 38.0783 | 77-53-2 | 9.97E+04±3.89E+03 | 8.16E+04±4.92E+03 | 0.82 |
| **Diterpenoid** |  |  |  |  |  |
| 6,10,14-trimethyl-2-Pentadecanone | 43.243 | 502-69-2 | 1.61E+05±6.93E+03 | 2.29E+05±6.87E+03 | 1.42 |
| **Aromatic derivatives** |  |  |  |  |  |
| 2,5-Pyrrolidinedione, 1-(benzoyloxy)- | 18.0605 | 23405-15-4 | 2.98E+05±3.51E+03 | 4.55E+05±1.41E+04 | 1.53 |
| Phenylethyl Alcohol | 23.5849 | 1960-12-8 | 3.53E+06±8.96E+04 | 4.04E+06±1.62E+05 | 1.14 |
| 2-Phenylethanamidine | 24.5312 | 5504-24-5 | 9.22E+04±3.50E+03 | 1.47E+05±5.89E+03 | 1.60 |
| Methyl salicylate | 26.3278 | 119-36-8 | 1.45E+07±8.66E+05 | 1.40E+07±3.29E+05 | 0.96 |
| 2,5-Cyclohexadiene-1,4-dione, | 33.9991 | 719-22-2 | 1.05E+05±5.05E+03 | 1.64E+05±6.57E+03 | 1.56 |
| 2,6-bis(1,1-dimethylethyl)- |  |  |  |  |  |
| 2,6-bis(1,1-dimethylethyl)-1,4-Benzenediol | 33.9995 | 2444-28-2 | 5.14E+04±2.52E+03 | 3.21E+04±1.44E+03 | 0.62 |
| Phenol, 2,4-bis(1,1-dimethylethyl)- | 34.9554 | 96-76-4 | 1.01E+06±6.64E+04 | 1.21E+06±3.51E+04 | 1.20 |
| Phthalic acid, butyl undecyl ester | 43.6881 | 1000308-91-2 | 1.02E+05±4.77E+03 | 1.75E+05±6.83E+03 | 1.72 |
| **Lipid derivative** |  |  |  |  |  |
| R-(-)-Cyclohexylethylamine | 4.9692 | 5913-13-3 | 1.89E+06±7.19E+04 | 2.00E+06±4.01E+04 | 1.06 |
| Hexanal | 11.2595 | 66-25-1 | 2.90E+05±1.33E+04 | 4.94E+05±1.83E+04 | 1.70 |
| (E)-2-Hexenal | 13.3064 | 6728-26-3 | 5.25E+06±2.26E+05 | 6.83E+06±1.43E+05 | 1.30 |
| Heptanal | 15.3028 | 111-71-7 | 1.32E+05±8.06E+03 | 2.21E+05±4.19E+03 | 1.67 |
| 1-Octen-3-ol | 18.4809 | 3391-86-4 | 1.09E+05±5.11E+03 | 4.58E+04±1.65E+03 | 0.42 |
| 3-Hexen-1-ol, acetate, (Z)- | 19.4015 | 3681-71-8 | 1.47E+07±5.60E+05 | 1.45E+07±4.50E+05 | 0.99 |
| 2-Hexen-1-ol, acetate | 19.7617 | 10094-40-3 | 7.71E+04±3.16E+03 | 3.12E+04±1.28E+03 | 0.40 |
| 2-ethyl-1-Hexanol | 20.3575 | 104-76-7 | 8.67E+05±3.04E+04 | 7.48E+05±3.44E+04 | 0.86 |
| 1-methylene-4-(1-methylethenyl)-Cyclohexane, | 21.5836 | 499-97-8 | 1.23E+05±4.54E+03 | 1.44E+05±7.18E+03 | 1.17 |
| 1-Octanol | 21.9239 | 111-87-5 | 4.28E+05±2.10E+04 | 5.35E+05±7.05E+02 | 1.25 |
| Ethyl 2-(5-methyl-5-vinyltetrahydrofuran | 22.0588 | 1000373-80-3 | 5.33E+06±2.56E+05 | 6.27E+06±2.45E+05 | 1.18 |
| -2-yl)propan-2-yl carbonate |  |  |  |  |  |
| Nonanal | 23.1748 | 124-19-6 | 1.60E+06±4.68E+04 | 2.27E+06±7.24E+04 | 1.42 |
| Cyclohexane, 2-ethenyl-1,1-dimethyl-3-methylene- | 23.4348 | 95452-08-7 | 7.35E+05±3.60E+04 | 1.59E+05±5.88E+03 | 0.22 |
| (6,6-Dimethylbicyclo[3.1.1]hept- | 24.1056 | 1000373-80-4 | 8.99E+04±4.05E+03 | 5.93E+04±1.29E+02 | 0.66 |
| 2-ene-2-yl)methyl ethyl carbonate |  |  |  |  |  |
| Myrtenyl acetate | 24.1058 | 1079-01-2 | 1.03E+05±4.42E+03 | 1.31E+05±5.91E+03 | 1.28 |
| Cyclopropane, trimethyl(2-methyl-1-propenylidene)- | 24.4161 | 14803-30-6 | 8.66E+04±3.98E+03 | 6.57E+04±3.22E+03 | 0.76 |
| 7-Oxabicyclo[4.1.0]heptane, 3-oxiranyl- | 25.0766 | 106-87-6 | 3.43E+04±1.58E+03 | 3.25E+04±1.62E+03 | 0.95 |
| 1-Nonanol | 25.3869 | 143-08-8 | 4.83E+05±1.88E+04 | 6.40E+05±7.04E+03 | 1.33 |
| 2H-Pyran-3-ol, 6-ethenyltetrahydro-2,2,6-trimethyl- | 25.6322 | 14049-11-7 | 6.59E+05±2.70E+04 | 3.42E+05±5.48E+03 | 0.52 |
| cis-3-Hexenyl iso-butyrate | 25.8073 | 41519-23-7 | 1.21E+05±5.68E+03 | 2.46E+05±9.59E+03 | 2.04 |
| Butanoic acid, 3-hexenyl ester, (E)- | 25.8171 | 53398-84-8 | 3.57E+05±1.72E+04 | 3.20E+05±6.71E+03 | 0.89 |
| Decanal | 26.5329 | 112-31-2 | 8.53E+05±3.84E+04 | 1.15E+06±1.48E+04 | 1.35 |
| Bicyclo[2.2.1]heptane-2,5-diol, | 27.6135 | 10359-41-8 | 1.99E+05±7.77E+03 | 1.15E+05±3.35E+03 | 0.58 |
| 1,7,7-trimethyl-, (2-endo,5-exo)- |  |  |  |  |  |
| 3-Heptadecen-5-yne, (Z)- | 27.7686 | 74744-55-1 | 1.44E+05±5.63E+03 | 1.35E+05±6.07E+03 | 0.94 |
| Cyclopentanecarboxylic acid, 2-phenylethyl ester | 28.1041 | 1000282-43-2 | 6.00E+05±2.70E+04 | 6.30E+05±2.90E+04 | 1.05 |
| 4-Hexen-1-ol, 2-ethenyl-2,5-dimethyl- | 28.3091 | 50598-21-5 | 8.92E+04±4.37E+03 | 1.18E+05±5.91E+03 | 1.33 |
| Cyclohexene, 1-(3-ethoxy-1-propenyl)-, (Z)- | 28.6647 | 51149-78-1 | 5.20E+04±2.50E+03 | 8.20E+04±2.38E+03 | 1.58 |
| Ethanol, 2-(3,3-dimethylbicyclo | 28.6693 | 2226-05-3 | 1.06E+05±4.89E+03 | 4.60E+04±2.12E+03 | 0.43 |
| [2.2.1]hept-2-ylidene)- |  |  |  |  |  |
| Undecanal | 29.6102 | 112-44-7 | 3.61E+04±1.52E+03 | 9.41E+04±4.33E+03 | 2.61 |
| 6,11-Dimethyl-2,6,10-dodecatrien-1-ol | 30.0009 | 1000196-53-3 | 3.11E+05±1.66E+04 | 4.04E+05±8.48E+03 | 1.30 |
| 6,9,12-Octadecatrienoic acid, | 30.9516 | 77509-03-6 | 3.90E+04±1.99E+03 | 6.73E+04±2.09E+03 | 1.73 |
| phenylmethyl ester, (Z,Z,Z)- |  |  |  |  |  |
| 13-Tetradece-11-yn-1-ol | 31.3371 | 1000131-00-4 | 1.69E+05±1.21E+04 | 2.16E+05±4.05E+03 | 1.28 |
| Hexanoic acid, 4-hexen-1-yl ester | 31.5770 | 88552-98-1 | 1.70E+05±6.45E+03 | 1.27E+05±4.95E+03 | 0.75 |
| Cyclopropane, 2-(1,1-dimethyl | 31.6871 | 81051-15-2 | 5.66E+04±2.55E+03 | 5.18E+04±1.81E+03 | 0.92 |
| -2-propenyl)-1,1-dimethyl- |  |  |  |  |  |
| 1-Hydroxy-1,7-dimethyl-4-isopropyl | 32.1278 | 72120-50-4 | 1.01E+05±4.23E+03 | 1.59E+05±4.62E+03 | 1.58 |
| -2,7-cyclodecadiene |  |  |  |  |  |
| 1-Octadecyne | 32.4380 | 629-89-0 | 3.94E+04±1.50E+03 | 8.21E+04±2.55E+03 | 2.08 |
| cubedol | 34.1994 | 1000374-15-9 | 2.83E+04±1.36E+03 | 7.87E+04±3.54E+03 | 2.78 |
| 4-epi-cubedol | 35.4857 | 1000374-16-0 | 1.26E+05±7.08E+03 | 1.50E+05±4.64E+03 | 1.18 |
| Naphthalene, 1,2,3,4-tetrahydro-1,6- | 35.6405 | 483-77-2 | 7.18E+04±3.30E+03 | 7.68E+04±3.69E+03 | 1.07 |
| dimethyl-4-(1-methylethyl)-, (1S-cis)- |  |  |  |  |  |
| Pentanoic acid, 2,2,4-trimethyl-3- | 36.9820 | 1000140-77-5 | 4.10E+04±1.09E+03 | 6.52E+04±3.20E+03 | 1.59 |
| carboxyisopropyl, isobutyl ester |  |  |  |  |  |
| Tricyclo[3.1.0.0(2,4)]hexane, 3,6 | 37.1060 | 1000150-21-2 | 6.19E+04±2.85E+03 | 1.14E+05±4.43E+03 | 1.84 |
| -diethyl-3,6-dimethyl-, trans- |  |  |  |  |  |
| 8-Dodecen-1-ol, acetate, (Z)- | 37.4527 | 28079-04-1 | 3.17E+04±1.24E+03 | 3.68E+04±1.58E+03 | 1.16 |
| E,E-10,12-Hexadecadienal | 39.4388 | 1000130-85-8 | 4.08E+04±1.96E+03 | 7.69E+04±3.69E+03 | 1.89 |
| 1H-Indene, 5-butyl-6-hexyloctahydro- | 43.1425 | 55044-36-5 | 5.11E+04±2.35E+03 | 9.18E+04±3.95E+03 | 1.80 |
| **Other compounds** |  |  |  |  |  |
| Oxime-, methoxy-phenyl-_ | 14.9876 | 1000222-86-6 | 1.44E+05±6.61E+03 | 2.45E+05±9.05E+03 | 1.70 |
