# Supplementary material for: Effects of vitro sucrose on quality components of tea plants (Camellia sinensis) based on transcriptomic and metabolic analysis
Source: BMC Plant Biol. 2018 Jun 18;18:121. doi: 10.1186/s12870-018-1335-0 (PMC6007066; doi:10.1186/s12870-018-1335-0)
Supplement: Supplementary file 2 — Table S2. Effects of sucrose on the expression of genes related to aroma. (DOCX 26 kb) [file 12870_2018_1335_MOESM2_ESM.docx]

| Gene  type | Gene  ID | Homologue protein  and accession | Size  (AA) | Query coverage  (%) | E-value | Identity  (%) | Control-vs-Suc  (log2 ratio) | |
| --- | --- | --- | --- | --- | --- | --- | --- | --- |
|  |  |  |  |  |  |  | 2ndD | 14thD |
| Terpenoid backbone  biosynthesis | CL12062.Contig1 | [HMG-CoA reductase2](https://blast.ncbi.nlm.nih.gov/Blast.cgi#alnHdr_619498810) ([AHY03303.1](https://www.ncbi.nlm.nih.gov/protein/619498810?report=genbank&log$=prottop&blast_rank=1&RID=WVR6RMEX015)) | 434 | 99 | 0 | 96 | 1.71 | -0.98 |
|  | CL12062.Contig2 | HMG-CoA reductase (AGB06349.1) | 425 | 100 | 0 | 98 | 0.18 | 0.015 |
|  | Unigene57617 | 1-deoxy-D-xylulose-5-phosphate synthase ([AJB84618.1](https://www.ncbi.nlm.nih.gov/protein/743066319?report=genbank&log$=protalign&blast_rank=1&RID=WVSDDT47014)) | 140 | 100 | 6e-96 | 100 | 2.66 | 1.96 |
|  | Unigene46601 | 1-deoxy-D-xylulose-5-phosphate  reductoisomerase ([AKE33276.1](https://www.ncbi.nlm.nih.gov/protein/814603307?report=genbank&log$=protalign&blast_rank=1&RID=WVSDDT47014)) | 43 | 100 | 3e-12 | 81 | 1.29 | -0.22 |
|  | CL5960.Contig2 | 1-deoxy-D-xylulose-5-phosphate synthase ([XP_017185144.1](https://www.ncbi.nlm.nih.gov/protein/1039926373?report=genbank&log$=prottop&blast_rank=1&RID=WVSDDT47014)) | 55 | 100 | 1e-26 | 80 | 3.37 | -0.86 |
| Monoterpene and | CL1850.Contig3 | Linalool synthase[*Actinidia arguta*]（ADD81294.1） | 568 | 100 | 0 | 64 | 0.39 | -0.72 |
| Sesquiterpenoid | CL634.Contig3 | (*E*)-β-ocimene synthase[*Vitis vinifera*]（ADR74204.1） | 564 | 100 | e-171 | 54 | 1.61 | -0.20 |
| synthase | Unigene46443 | alpha-farnesene synthase-like*[Hevea brasiliensis]*（[XP_021655200.1](https://www.ncbi.nlm.nih.gov/protein/XP_021655200?report=genbank&log$=protalign&blast_rank=4&RID=58KGM5A8014)） | 182 | 100 | 1e-127 | 99 | 2.21 | 2.14 |
|  | Unigene9305 | (E)-nerolidol synthase [*Camellia sinensis*] ([ALO81817.1](https://www.ncbi.nlm.nih.gov/protein/953768617?report=genbank&log$=protalign&blast_rank=1&RID=WVTH88E0014)) | 129 | 99 | 4e-81 | 91 | 3.58 | -1.72 |
|  | Unigene58074 | Myrcene synthase[*Vitis vinifera*]（XP_002266808.1） | 564 | 100 | 0 | 67 | 1.61 | -0.20 |
|  | CL5257.Contig2 | (*R*)-limonene synthase[*Ricinus communis*]（XP_002515689.1） | 509 | 100 | 0 | 68 | 1.45 | -0.32 |
|  | CL634.Contig3 | (–)–α-terpineol synthase[*Vitis vinifera*]（NP_001268216.1） | 564 | 100 | 0 | 66 | 1.61 | -0.20 |
|  | Unigene36257 | (+)-α-phellandrene synthase[*Vitis vinifera*]（ADR74201.1） | 564 | 100 | 0 | 54 | -3.13 | 1.90 |
| Diterpenoid biosynthesis | Unigene46267 | terpene synthase ( [AFE56211.1](https://www.ncbi.nlm.nih.gov/protein/380751740?report=genbank&log$=protalign&blast_rank=1&RID=WVTH88E0014)) | 467 | 100 | 0 | 91 | 2.78 | 1.03 |
| Triterpenoid biosynthesis | Unigene32959 | flavone synthase II( [ACH99109.1](https://www.ncbi.nlm.nih.gov/protein/204304434?report=genbank&log$=protalign&blast_rank=1&RID=WVTH88E0014)) | 534 | 100 | 0 | 99 | 1.04 | 0.38 |
| Lipoxygenase | CL4399.Contig2 | Lipoxygenase ( [ADO51752.1](https://www.ncbi.nlm.nih.gov/protein/308943877?report=genbank&log$=protalign&blast_rank=1&RID=WVTH88E0014)) | 211 | 100 | 5e-129 | 90 | -1.32 | -0.08 |
|  | CL4714.Contig2 | Lipoxygenase( [ADL41189.1](https://www.ncbi.nlm.nih.gov/protein/302566881?report=genbank&log$=protalign&blast_rank=1&RID=WVTH88E0014)) | 868 | 100 | 0 | 99 | -1.22 | -6.88 |
| β-Primeverosidase | CL11470.Contig2 | β-Primeverosidase（BAC78656.1 ） | 507 | 100 | 0 | 99 | 0.35 | -0.63 |

Table S2 Effects of sucrose on the expression of genes related to aroma.
