# Supplementary figures and images for: Effects of vitro sucrose on quality components of tea plants (Camellia sinensis) based on transcriptomic and metabolic analysis
Source: BMC Plant Biol. 2018 Jun 18;18:121. doi: 10.1186/s12870-018-1335-0 (PMC6007066; doi:10.1186/s12870-018-1335-0)

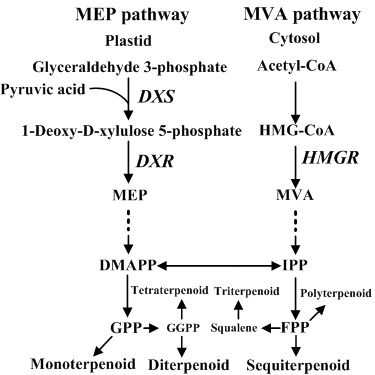

Supplement: Supplementary file 3 — Figure S1. The pathway of terpenoids biosynthesis. (TIF 412 kb) [file 12870_2018_1335_MOESM3_ESM.tif]

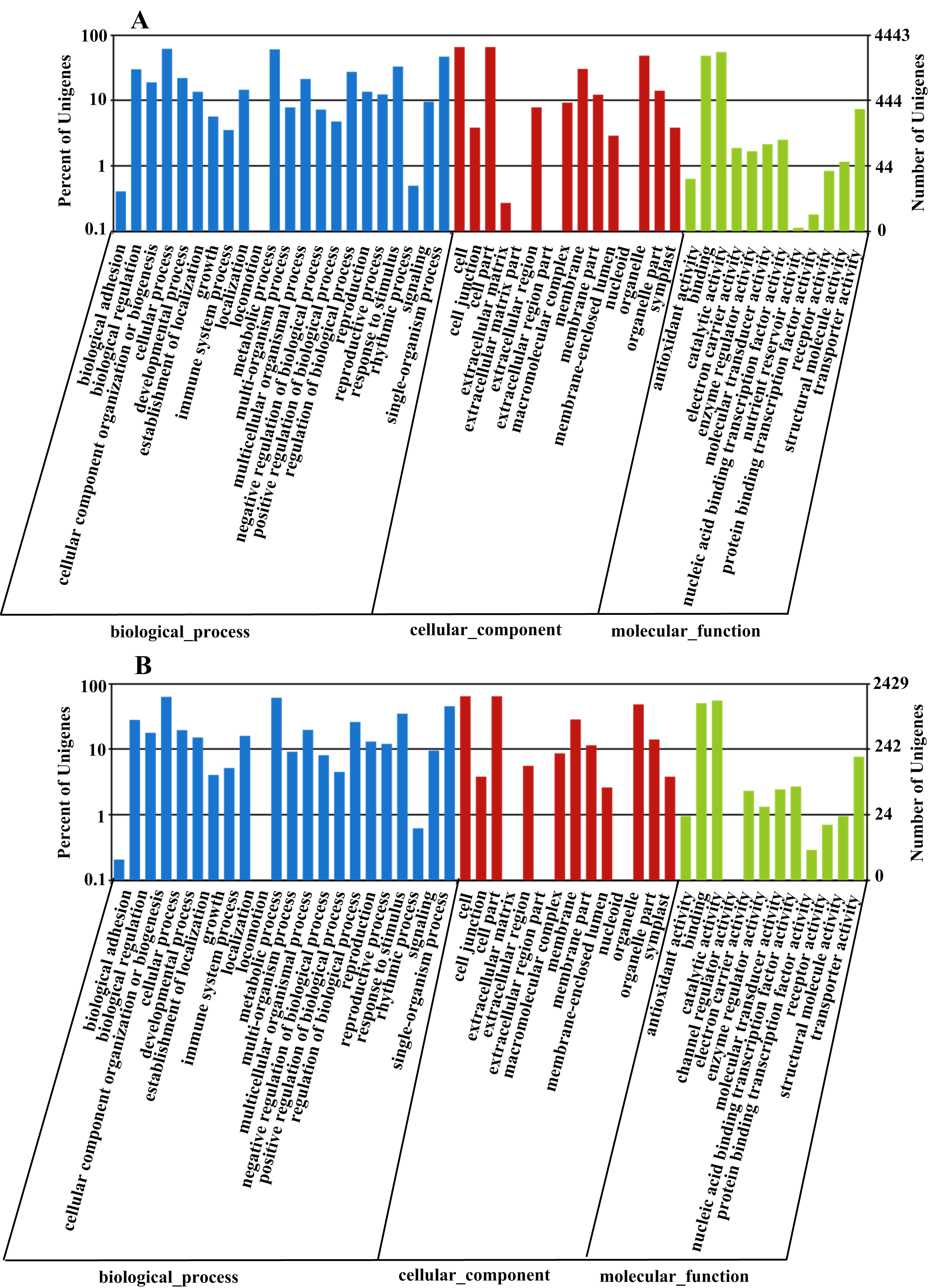

Supplement: Supplementary file 7 — Figure S2. GO functional classification of DEGs obtained from tea plants treated by sucrose after 2d (A) and 14d (B). Note: GO functions are showed on X-axis, the right Y-axis shows the number of DEGs which have the GO function, the left Y-axis shows the percentage of DEGs. (TIF 27083 kb) [file 12870_2018_1335_MOESM7_ESM.tif]

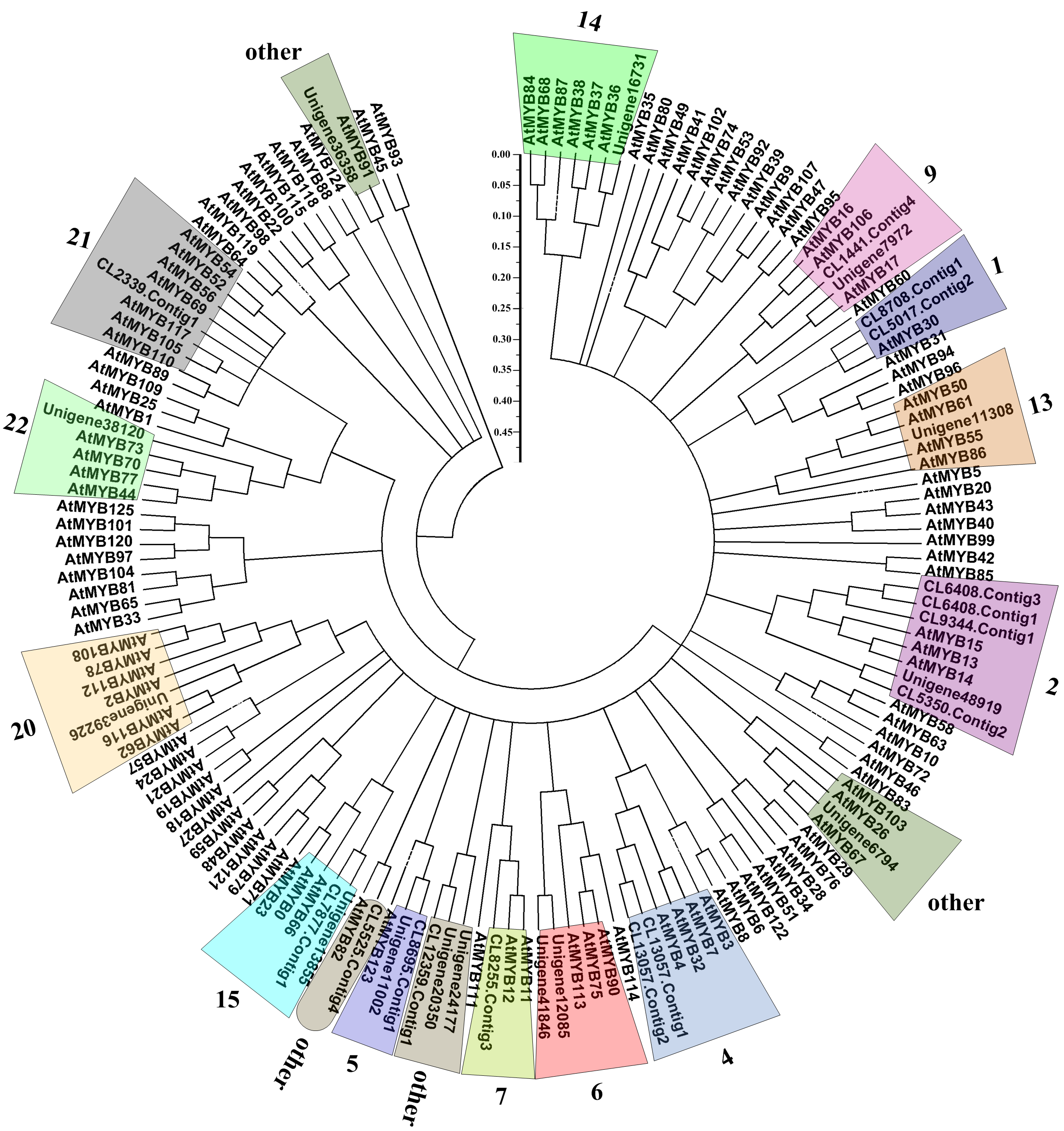

Supplement: Supplementary file 8 — Figure S3. Evolutionary relationships of DEGs belong to R2R3-MYB obtained from tea plants treated by sucrose. Note: The phylogenetic tree was constructed based on amino acid sequences using MEGA5 per the neighbor-joining method, digit indicates subgroup, other indicates DEGs are not grouped. (TIF 18963 kb) [file 12870_2018_1335_MOESM8_ESM.tif]

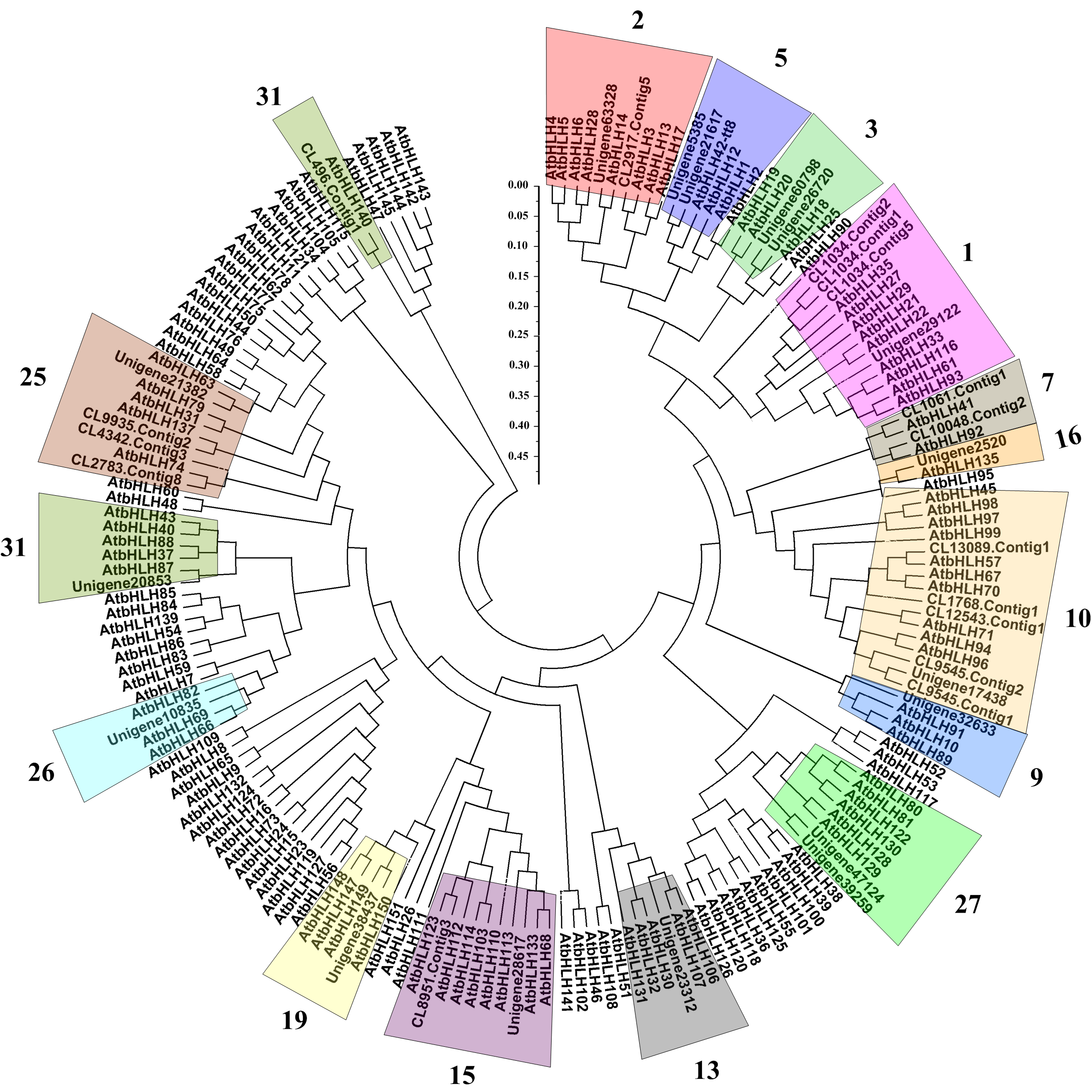

Supplement: Supplementary file 9 — Figure S4. Evolutionary relationships of DEGs belong to bHLH obtained from tea plants treated by sucrose. Note: The phylogenetic tree was constructed based on amino acid sequences using MEGA5 according to the neighbor-joining method, digit indicates subfamily. (TIF 16115 kb) [file 12870_2018_1335_MOESM9_ESM.tif]

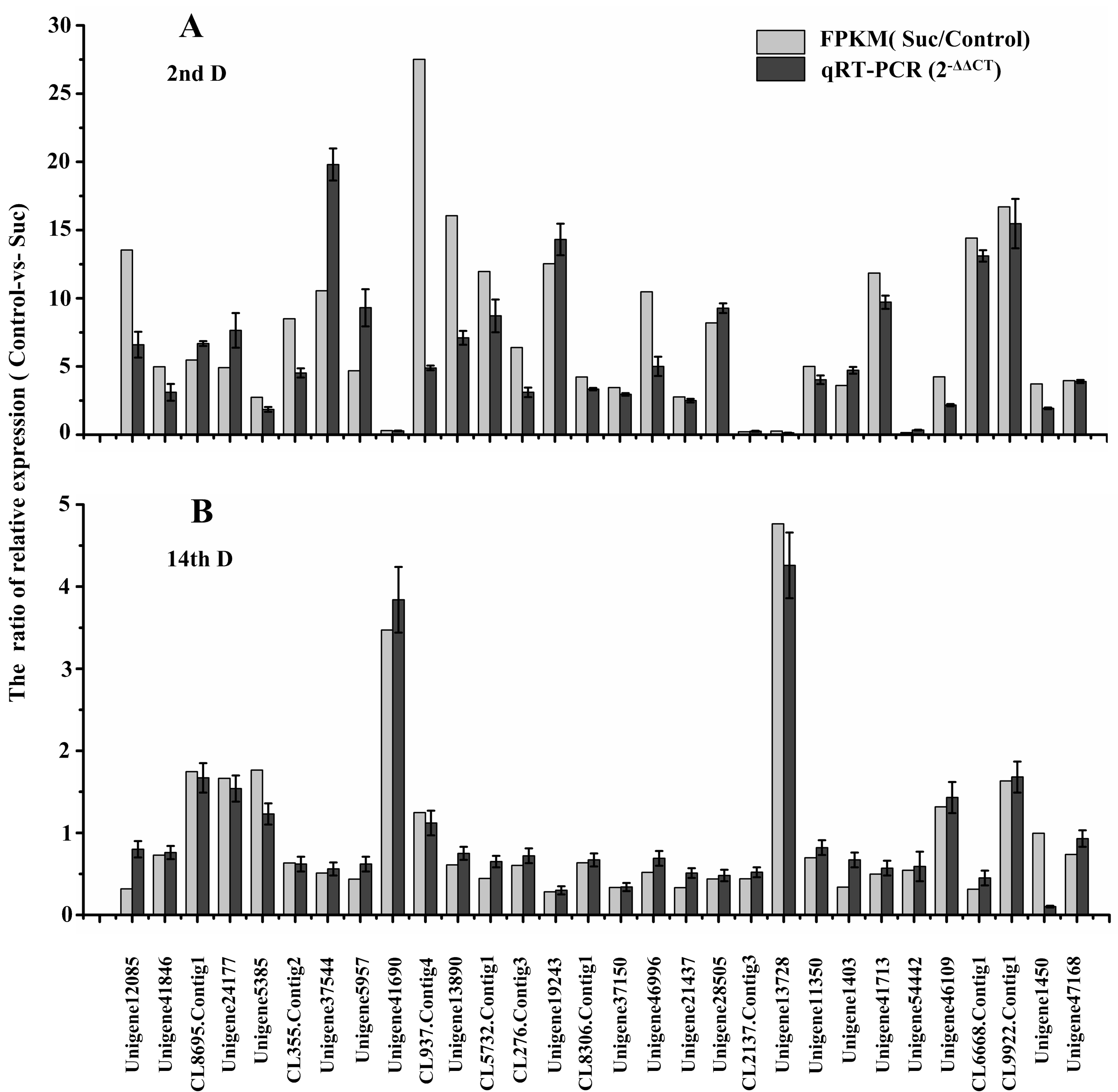

Supplement: Supplementary file 11 — Figure S5. Validation of DEGs obtained from tea plants treated by sucrose using qRT-PCR. A. DEGs obtained from tea plants treated by sucrose after 2d; B. DEGs obtained from tea plants treated by sucrose after 14d. Note: The data of qRT-PCR represents the mean value of three biological and three technical replicates. (TIF 16000 kb) [file 12870_2018_1335_MOESM11_ESM.tif]
