# Supplementary material for: Effects of vitro sucrose on quality components of tea plants (Camellia sinensis) based on transcriptomic and metabolic analysis
Source: BMC Plant Biol. 2018 Jun 18;18:121. doi: 10.1186/s12870-018-1335-0 (PMC6007066; doi:10.1186/s12870-018-1335-0)
Supplement: Supplementary file 4 — Table S3. Statistics of sequencing output. Note: Q20 percentage is the proportion of nucleotides with quality value larger than 20, N percentage is proportion of unknown nucleotides in clean reads, GC percentage is proportion of guanidine and cytosine nucleotides among total nucleotides. (DOCX 20 kb) [file 12870_2018_1335_MOESM4_ESM.docx]

Table S3. Statistics of sequencing-output.

| Samples | Total  Raw Reads | Total  Clean Reads | Total Clean Nucleotides (nt) | Q20 percentage | N percentage | GC percentage |
| --- | --- | --- | --- | --- | --- | --- |
| 2nd D Control | 67,703,548 | 64,029,390 | 5,762,645,100 | 97.97% | 0.00% | 43.90% |
| 2nd D Suc | 69,535,792 | 65,816,856 | 5,923,517,040 | 98.08% | 0.00% | 43.85% |
| 14th D Control | 55,340,090 | 52,794,250 | 4,751,482,500 | 98.67% | 0.00% | 44.84% |
| 14th D Suc | 57,642,524 | 54,928,322 | 4,943,548,980 | 98.69% | 0.00% | 44.86% |
| All | 250,221,954 | 237,568,818 | 21,381,193,620 |  |  |  |

Note: Q20 percentage is the proportion of nucleotides with quality value larger than 20, N percentage is proportion of unknown nucleotides in clean reads, GC percentage is proportion of guanidine and cytosine nucleotides among total nucleotides.

GC percentage is proportion of guanidine and cytosine nucleotides among total nucleotides.
