# Supplementary material for: Effects of vitro sucrose on quality components of tea plants (Camellia sinensis) based on transcriptomic and metabolic analysis
Source: BMC Plant Biol. 2018 Jun 18;18:121. doi: 10.1186/s12870-018-1335-0 (PMC6007066; doi:10.1186/s12870-018-1335-0)
Supplement: Supplementary file 5 — Table S4. Statistics of assembly quality. Note: Total Consensus Sequences represents the all assembled Unigenes, Distinct Clusters represents the cluster Unigenes; the same cluster contains some highly similar (more than 70%) Unigenes and these may come from same gene or homologous gene, Distinct Singletons represents Unigenes from a single gene. (DOCX 21 kb) [file 12870_2018_1335_MOESM5_ESM.docx]

Table S4. Statistics of assembly quality.

|  | Sample | Total Number | Total Length(nt) | Mean Length(nt) | N50  Length(nt) | Total Consensus | Distinct Clusters | Distinct Singletons |
| --- | --- | --- | --- | --- | --- | --- | --- | --- |
| Contig | 2nd D Control | 168,881 | 60,585,976 | 359 | 647 |  |  |  |
|  | 2nd D Suc | 180,566 | 64,694,733 | 358 | 645 |  |  |  |
|  | 14th D Control | 148,983 | 55,309,125 | 371 | 713 |  |  |  |
|  | 14th D Suc | 155,025 | 56,607,723 | 365 | 680 |  |  |  |
| Unigene | 2nd D Control | 110,061 | 89,988,747 | 818 | 1626 | 110,061 | 39,067 | 70,994 |
|  | 2nd D Suc | 117,685 | 97,098,794 | 825 | 1637 | 117,685 | 41,977 | 75,708 |
|  | 14th D Control | 97,255 | 82,798,459 | 851 | 1669 | 97,255 | 35,224 | 62,031 |
|  | 14th D Suc | 102,223 | 86,553,156 | 847 | 1655 | 102,223 | 37,191 | 65,032 |
|  | All | 118,843 | 144,066,247 | 1,212 | 1,999 | 118,843 | 53,957 | 64,886 |

Note: Total Consensus Sequences represents the all assembled Unigenes; Distinct Clusters represents the cluster Unigenes; the same cluster contains some high similar (more than 70%) Unigenes and these Unigenes may come from same gene or homologous gene; Distinct Singleto
