# Supplementary material for: Effects of vitro sucrose on quality components of tea plants (Camellia sinensis) based on transcriptomic and metabolic analysis
Source: BMC Plant Biol. 2018 Jun 18;18:121. doi: 10.1186/s12870-018-1335-0 (PMC6007066; doi:10.1186/s12870-018-1335-0)
Supplement: Supplementary file 6 — Table S5. Summary of Unigenes annotated to six databases. (DOCX 20 kb) [file 12870_2018_1335_MOESM6_ESM.docx]

Table S5. Summary of Unigenes annotated to six databases.

| Annotated databases | Number of annotated Unigenes | Percent of annotated Unigenes |
| --- | --- | --- |
| NR | 76,858 | 64.67 |
| NT | 74,546 | 62.73 |
| Swiss-Prot | 51,731 | 43.53 |
| KEGG | 47,655 | 40.10 |
| COG | 32,183 | 27.08 |
| GO | 60,517 | 50.92 |
| all-annotated Unigenes | 82,459 | 69.39 |
| all-Unigenes | 118,843 |  |
