# Supplementary material for: Effects of vitro sucrose on quality components of tea plants (Camellia sinensis) based on transcriptomic and metabolic analysis
Source: BMC Plant Biol. 2018 Jun 18;18:121. doi: 10.1186/s12870-018-1335-0 (PMC6007066; doi:10.1186/s12870-018-1335-0)
Supplement: Supplementary file 12 — Table S7. Primers used for qRT-PCR and detailed information regarding the selected DEGs. Note: “↑” indicates significant up-regulation; “–”no difference; “↓”indicates significant down-regulation. (DOCX 39 kb) [file 12870_2018_1335_MOESM12_ESM.docx]

Table S7. Primers used for qRT-PCR and detailed information regarding the selected DEGs.

| Gene ID  ( Gene name ) | Swissprot-annotation | The ratio of FPKM Suc/Control （2^-ΔΔCT^） | | Primers sequences (5'-3') for qRT-PCR |
| --- | --- | --- | --- | --- |
|  |  | 2 d | 14 d |  |
| *GAPDH* | glyceraldehyde-3-phosphate dehydrogenase |  |  | F:TTGGCATCGTTGAGGGTCT  R:CAGTGGGAACACGGAAAGC |
| *Unigene2938_All* | Phenylalanine ammonia-lyase (CsPAL_A_) | 4.35↑ (4.51±0.98 ↑) | 1.54– (1.59±0.60 –) | F:CCGTTCAAGCAAGCAGT  R:ACATTGTAGCCCTCGTAGA |
| *CL586.Contig5_All* | Phenylalanine ammonia-lyase (CsPAL_B_) | 2.49 ↑ | 3.41 ↑ |  |
| *CL586.Contig6_All* | Phenylalanine ammonia-lyase (CsPAL_C_) | 4.02 ↑ | 1.30 – |  |
| *CL2080.Contig3_All* | Phenylalanine ammonia-lyase (CsPAL_D_) | 6.90 ↑ | 1.23 – |  |
| *CL586.Contig1_All* | Phenylalanine ammonia-lyase (CsPAL_E_) | 4.57 ↑ | 1.28 – |  |
| *CL586.Contig16_All* | Phenylalanine ammonia-lyase (CsPAL_F_) | 1.88 – | 1.94 – |  |
| *CL6859.Contig3_All* | Trans-cinnamate 4-monooxygenase (CsC4H_A_) | 2.58 ↑ (4.22±1.78↑) | 1.21 – (0.88±0.27 –) | F:CGATAGAATGGGGCATAGCA  R:TGGAGGTAGGGGAGTTTGTAGG |
| *CL6859.Contig2_All* | Trans-cinnamate 4-monooxygenase (CsC4H_B_) | 3.63 ↑ | 1.10 – |  |
| *CL6859.Contig1_All* | Trans-cinnamate 4-monooxygenase (CsC4H_C_) | 3.51 ↑ | 1.17 – |  |
| *CL7152.Contig1_All* | 4-coumarate--CoA ligase (Cs4CL_A_) | 2.10 ↑ | 0.88 – |  |
| *CL12953.Contig1_All* | 4-coumarate--CoA ligase (Cs4CL_B_) | 3.22 ↑ | 1.18 – |  |
| *Unigene43035_All* | 4-coumarate--CoA ligase (Cs4CL_C_) | 3.11 ↑ (5.25±1.02 ↑) | 1.17 – (0.94±0.49 –) | F:AGCGGCATAACTATTACCACAGC  R:TCCCGACGAATAAGGTAGCG |
| *Unigene27917_All* | Chalcone synthase (CsCHS_A_) | 2.72 ↑ (6.49±1.25 ↑) | 1.33 – (1.95±0.51 –) | F:TGAAGGACCTGCCACGGTTATG  R:GCCTTATGCTCGCTGTTTGT |
| *CL4837.Contig1_All* | Chalcone synthase (CsCHS_B_) | 2.28 ↑ | 1.24 – |  |
| *Unigene26537_All* | Chalcone synthase (CsCHS_C_) | 2.66 ↑ | 1.09 – |  |
| *CL5073.Contig1_All* | Chalcone--flavonone isomerase (CsCHI_A_) | 1.92 – | 1.06 – |  |
| *CL12084.Contig2_All* | Chalcone--flavonone isomerase (CsCHI_B_) | 2.41 ↑ (2.87±0.63 ↑) | 1.49 – (0.90±0.09 –) | F:AAGCACCAACAAAACCTACCA  R:AAACACCATTACCAATCGCA |
| *Unigene18276_All* | Chalcone--flavonone isomerase (CsCHI_C_) | 2.05 ↑ | 1.17 – |  |
| *Unigene632_All* | Naringenin,2-oxoglutarate 3-dioxygenase (CsF3H_A_) | 2.49 ↑ (9.03±2.51 ↑) | 1.50 – (1.90±0.45 –) | F:ACAACAACGCTTACGGCTCTC  R:AAACCCCCCAACCTTCACA |
| *CL781.Contig3_All* | Naringenin,2-oxoglutarate 3-dioxygenase (CsF3HB) | 2.89 ↑ | 1.41 – |  |
| *Unigene38370_All* | Flavonoid 3'-monooxygenase (CsF3^'^ H_A_) | 2.43 ↑ (8.05±0.5 ↑) | 1.67 – (1.09±0.4 –) | F:CCGACAGGAGGAGATAGCGATA  R:GGAAGCAACACCTTGGGGAT |
| *Unigene18200_All* | Flavonoid 3'-monooxygenase (CsF3^'^H_B_) | 2.63 ↑ | 1.59 – |  |
| *Unigene47986_All* | Flavonoid 3'-monooxygenase (CsF3^'^H_C_) | 1.80 – | 0.89 – |  |
| *Unigene40628_All* | Flavonoid 3',5'-hydroxylase (CsF3^'^5^'^H_A_) | 4.14 ↑ | 1.04 – |  |
| *Unigene42162_All* | Flavonoid 3',5'-hydroxylase (CsF3^'^5^'^H_B_) | 5.22 ↑ (9.44±1.36 ↑) | 1.35 – (1.61±0.60 –) | F:CAAAAAAGTCTTAGCCGTCGC  R:CTCTCAATCCCTTGTAAGTCCATC |
| *Unigene43065_All* | Flavonol synthase (CsFLS_A_) | 2.37 ↑ | 1.27 – |  |
| *CL5193.Contig5_All* | Flavonol synthase (CsFLS_B_) | 1.60 – | 1.97 – |  |
| *Unigene47836_All* | Flavonol synthase (CsFLS_C_) | 1.75 – | 1.14 – |  |
| *Unigene23111_All* | Dihydroflavonol-4-reductase (CsDFR_A_) | 2.65 ↑ (2.66±0.05 ↑) | 0.91 – (0.88±0.16 –) | F:ATTCATCGGCTCGTGGC  R:TTCTCAGGGTCCTTAGACTCAA |
| *Unigene36268_All* | Dihydroflavonol-4-reductase (CsDFR_B_) | 7.52 ↑ | 0.83 – |  |
| *CL4050.Contig5_All* | Dihydroflavonol-4-reductase (CsDFR_C_) | 0.55 – | 0.72 – |  |
| *CL4050.Contig4_All* | Dihydroflavonol-4-reductase (CsDFR_D_) | 0.55 – | 1.43 – |  |
| *Unigene22228_All* | anthocyanidin synthase (CsANSA) | 3.14 ↑ | 0.77 – |  |
| *CL10930.Contig2_All* | anthocyanidin synthase (CsANS_B_) | 5.19 ↑ | 1.03 – |  |
| *CL6364.Contig2_All* | Leucoanthocyanidin reductase (CsLAR_A_) | 2.56 ↑ | 1.22 – |  |
| *Unigene18021_All* | Leucoanthocyanidin reductase (CsLAR_B_) | 0.94 – | 1.59 – |  |
| *Unigene23415_All* | Leucoanthocyanidin reductase (CsLAR_C_) | 5.04 ↑ (9.61±1.48 ↑) | 0.94 – (0.74±0.38 –) | F:TCAGAGTTTGGACATGACGTGG  R:CTGACTCCTCTATCAACCTCCG |
| *CL1300.Contig2_All* | anthocyanidin reductase (CsANR_A_) | 2.74 ↑ (16.19±2.75 ↑) | 1.41 – (1.22±0.26 –) | F:GAGTACTTCAAGGCTAAGGGGAT  R:CAAGCAAACCAAGCAAAACC |
| *CL1300.Contig1_All* | anthocyanidin reductase (CsANR_B_) | 2.26 ↑ | 1.28 – |  |
| *Unigene12085* | Transcription factor MYB75 | 13.54 ↑ (6.60±0.95↑) | 0.32 ↓ (0.80±0.1–) | F:GCATCATTGCTAGGGGATTGTG R:GTTGTAAGTTCTTCAGGCAGCC |
|  |  |  |  |  |
| *Unigene41846* | Transcription factor MYB90 | 4.98 ↑ (3.11±0.62↑) | 0.73 – (0.76±0.08 –) | F:CGAACAAGAGACAACCTTAGC R:GTCACTCCAATCACTCTGGTCT |
|  |  |  |  |  |
| *CL8695.Contig1* | Anthocyanin regulatory C1 protein | 5.47 ↑ (6.68±0.18 ↑) | 1.75 – (1.67±0.18 –) | F:CCTAGTCCGTACCAAGGCATCA R:ACAGTGTCAGTAGTGTCGCCT |
|  |  |  |  |  |
| *Unigene24177* | Myb-related protein 308 | 4.91 ↑ (7.65±1.27 ↑) | 1.66 – (1.54±0.16 –) | F:GCCCATTAGGATCAAGTCTTCG R:GAGGTCTGAAGACTCTGAACCA |
|  |  |  |  |  |
| *Unigene5385* | Transcription factor TT8 | 2.74 ↑ (1.86±0.17 –) | 1.76 – (1.23±0.13 –) | F:ATGAACCCCAGCTACCTACTC  R:TGGGAACCGAGAAGTGGAGTC |
|  |  |  |  |  |
| *CL355.Contig2* | UDP-glycosyltransferase 75D1 | 8.50↑ (4.53±0.34↑) | 0.63– (0.62±0.09–) | F:GTGGGTCATAAGGGCTACAGA R:GACCACACCACGGCACTATCA |
|  |  |  |  |  |
| *Unigene37544* | ABC transporter C family member 3 | 10.55 ↑ (19.81±1.17 ↑) | 0.51 – (0.56±0.08 –) | F:CGTGGACACTGACATTCCTTAC R:AAAGTGGTGGAGGATTGGAGC |
|  |  |  |  |  |
| *Unigene5957* | Auxin response factor 5 | 4.68↑(9.30±1.36↑) | 0.44↓ (0.62±0.09–) | F:AGGCTACGACTATGACACAGG R:CACTTGAGTGCTTGACCCGTAT |
|  |  |  |  |  |
| *Unigene41690* | Auxin-induced protein 10A5 | 0.30 ↓ (0.28±0.02 ↓) | 3.47 ↑ (3.84±0.4 ↑) | F:GAAAGGGTGTCTGGCAATCAAG R:GTGGCAAGGGATGGTGATAGT |
|  |  |  |  |  |
| *CL937.Contig4* | Indole-3-acetic acid-induced protein ARG7 | 27.50 ↑ (4.89±0.17 ↑) | 1.25 – (1.12±0.15 –) | F:GAACCAGCCTTCATTCCAAGAC R:ATCCTCCCAGTTGAGCAGTGA |
|  |  |  |  |  |
| *Unigene13890* | Glucan endo-1,3-beta-D-glucosidase | 16.05 ↑ (7.10±0.50 ↑) | 0.61 – (0.75±0.08 –) | F:CCTGCTTCTCCCCAGACAATC R:GGTGATGAGAGCAGAGTTCTTG |
|  |  |  |  |  |
| *CL5732.Contig1* | Glucan endo-1,3-beta-glucosidase 1 | 11.96 ↑ (8.71±120 ↑) | 0.44 ↓ (0.65±0.07 –) | F:TCCCCGATTGGTTACTCTGTTC R:ACACCTCACTCTTTTCCCCCAC |
|  |  |  |  |  |
| *CL276.Contig3* | Histidine kinase 4 | 6.39 ↑ (3.11±0.35 ↑) | 0.60 – (0.72±0.09 –) | F:CTTTGAGAGCGAGTATGGTGGC R:CTCTACGGTTCACTCTGTTGTC |
|  |  |  |  |  |
| *Unigene19243* | Transcription repressor KAN | 12.53 ↑ (14.32±1.16 ↑) | 0.28 ↓ (0.30±0.05 ↓) | F:GGGTAGTTGTGGAAGGAATGGAG R:GAGTCCTCTCACATCCATCAACTG |
|  |  |  |  |  |
| *CL8306.Contig1* | Two-component response regulator ARR17 | 4.23 ↑ (3.34±0.09 ↑) | 0.64 – (0.67±0.08 –) | F:GTTTGGTGGGACATGGATGCT R:GTCTCCCAATCCCAAATACTCC |
|  |  |  |  |  |
| *Unigene37150* | Two-component response regulator ARR9 | 3.45 ↑ (2.95±0.09 ↑) | 0.33 ↓ (0.34±0.05 ↓) | F:TGCTAAGAAGAGTGAAGGGGTC R:GCTCCTCCTTCCAAGCACATAT |
|  |  |  |  |  |
| *Unigene46996* | Probable inactive leucine-rich repeat | 10.48 ↑ (5.01±0.71 ↑) | 0.52 – (0.69±0.09 –) | F:CACCTCTTCAATGCCACCACTC  R:TGAGGAGATTGGAGGATAGGTC |
|  | receptor-like protein kinase At1g66830 |  |  |  |
| *Unigene21437* | Leucine-rich repeat receptor-like protein | 2.77↑ (2.50±0.13↑) | 0.33↓ (0.51±0.06–) | F:GATGCGTTATCTTGGTCTCGG R:CTATTGCCGTGTTTGTGCTGC |
|  | CLAVATA2 |  |  |  |
| *Unigene28505* | Probably inactive leucine-rich repeat | 8.19 ↑ (9.27±0.35 ↑) | 0.44 ↓ (0.48±0.07 ↓) | F:GTCCGATGTCTACAGCCTTGG R:GGAGACGGATCAACACAATGC |
|  | receptor-like protein kinase IMK2 |  |  |  |
| *CL2137.Contig3* | G-type lectin S-receptor-like | 0.22 ↓ (0.26±0.03 ↓) | 0.44 ↓ (0.52±0.06 –) | F:GATGCTTATTATGCCTCGGGG R:GATTCACCACCACTCGCATTG |
|  | serine/threonine-protein kinase At5g24080 |  |  |  |
| *Unigene13728* | Xyloglucan endotransglucosylase/hydrolase | 0.26 ↓ (0.14±0.01 ↓) | 4.77 ↑ (4.26±0.4 ↑) | F:AGGACAGATTGGGTAACAGAGC R:GGCCTAGTCATAACCTTTCCTG |
|  | protein 22 |  |  |  |
| *Unigene11350* | Cyclin-D3-1 OS | 5.00 ↑ (4.03±0.32 ↑) | 0.70 – (0.82±0.09 –) | F:TGCTGCCAAAGTTGAGGAGAC |
|  |  |  |  | R:GAGTGGAGTCACAGGATTCA |
| *Unigene1403* | Probable carboxylesterase 2 | 3.61 ↑ (4.72±0.25 ↑) | 0.34 ↓ (0.67±0.09 –) | F:ACCCTCTTCCTCTGGCTTA |
|  |  |  |  | R:GCCTCAACACCTGCTTTCACC |
| *Unigene41713* | Scarecrow-like protein 28 | 11.85 ↑ (9.71±0.49 ↑) | 0.50 ↓ (0.57±0.09 –) | F:TCCAGAACTAATCCTCCAAGCCR:GAAGCATCCAAAGCCTGACATC |
|  |  |  |  |  |
| *Unigene54442* | Probable protein phosphatase 2C 58 | 0.15 ↓ (0.35±0.03 ↓) | 0.54 – (0.59±0.18 –) | F:CAAGAGCCAAGATGAGAGAGG R:GTCTTTCGGTCCCTTGATGATG |
|  |  |  |  |  |
| *Unigene46109* | Serine/threonine-protein kinase SRK2A | 4.24 ↑ (2.17±0.08 ↑) | 1.32 – (1.43±0.19 –) | F:GAAGAACTTGCCTCGGGAACT R:GCTCGCATGTGCCTGTTTAAC |
|  |  |  |  |  |
| *CL6668.Contig1* | F-box/LRR-repeat protein 17 | 14.41 ↑ (13.11±0.42 ↑) | 0.31 ↓ (0.45±0.09 ↓) | F:CTAAGTTTGTGGGGCTGTTCTG:GGR:GGGTTTTCCACAGCAGCAGAT |
|  |  |  |  |  |
| *CL9922.Contig1* | Pathogenesis-related leaf protein 6 | 16.70 ↑ (15.48±1.81 ↑) | 1.63 – (1.68±0.19 –) | F:CGACGGAGTGAACCTTTGGATA  R:GTTGTCCAATGTAGTTGCCTC G |
|  |  |  |  |  |
| *Unigene1450* | Phosphatidylinositol 4-phosphate 5-kinase 1 | 3.72 ↑ (1.93±0.06 –) | 1.00 – (0.10±0.01 ↓) | F:GGGAAGATGGTGTTCCTAAAGG  R:CTCTCAGCCAAACTACCCCTCC |
|  |  |  |  |  |
| *Unigene47168* | Sucrose synthase | 3.96 ↑ (3.90±0.12↑) | 0.74 – (0.93±0.1 –) | F:TGGAGACTTTCACTGAGGACG |
|  |  |  |  | R:GGCAACAAGGTTACCCTCACT |

# Note: “↑” indicates significant up-regulation; “–”no difference; “↓”indicates significant down-regulation.
